# The Anomalously High Boiling Point of Water Revisited

**DOI:** 10.1002/open.70316

**Published:** 2026-10-13

**Authors:** Lucas de Azevedo Santos, Siebe Lekanne Deprez, F. Matthias Bickelhaupt, Célia Fonseca Guerra

**Affiliations:** ^1^ Department of Chemistry and Pharmaceutical Sciences AIMMS Vrije Universiteit Amsterdam Amsterdam Netherlands; ^2^ Institute for Molecules and Materials Radboud University Nijmegen Netherlands; ^3^ Department of Chemical Sciences University of Johannesburg Johannesburg South Africa

**Keywords:** boiling point, bond theory, density functional calculations, hydrogen bonds, Kohn–Sham molecular orbital theory

## Abstract

Typically, boiling points increase with molecular weight because dispersion forces strengthen. However, H_2_O deviates from this trend, exhibiting a higher boiling point than its heavier group‐16 analogs. This anomaly is traditionally attributed to water’s larger dipole moment, which is thought to promote stronger electrostatic intermolecular hydrogen bonds. Yet, this textbook explanation lacks quantitative support. Here, alongside thermodynamic and structural effects, we identify a key microscopic, quantum chemical factor that explains trends in interaction strength, which in turn are associated with water’s abnormally high boiling point. Within the series of binary hydrides studied, Kohn–Sham molecular orbital theory reveals that the Pauli exclusion principle is an important factor behind water’s high boiling point, as it modulates intermolecular distances and, thereby, influences the strength of all attractive interactions, including Coulombic attraction. The compact H_2_O molecule experiences significantly less intermolecular Pauli repulsion, allowing for shorter intermolecular distances, enhancing all attractive forces, and resulting in elevated boiling points. These findings not only provide a rigorous, quantitative framework for understanding a fundamental property of water but also call for a critical re‐evaluation of conventional teaching and interpretation.

## Graphical abstract

Pairwise interactions between binary hydrides depend mainly on size, not dipole moment. Small hydrides experience less steric repulsion, bringing molecules closer together and strengthening hydrogen bonds, which explains their high boiling points.

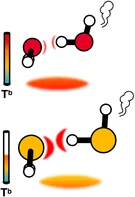

## 1 Introduction

In general chemistry books, the physical and chemical properties of small molecules are often described by classical mechanics. For example, the boiling point of substances generally increases with the molecular mass [1, 2, 3]. This trend is largely due to the fact that larger, heavier molecules contain more electrons, which facilitates the formation of temporary dipoles, enhancing the dispersion forces that hold molecules together [1, 2, 3]. Additionally, larger molecules have a greater surface area, allowing for more intermolecular interactions [1, 2, 3]. As a result, stronger dispersion forces acting over a larger area lead to more stabilizing interactions between molecules, increasing the energy required to separate them and, thus, raising their boiling points.

An increase in the boiling point is observed as the molecular mass of the binary hydrides of group‐16 elements increases as one moves down the group in the periodic table. For instance, at 1 atm, the boiling point rises from approximately −60 °C for H_2_S to around −41 °C for H_2_Se (Figure 1) [1, 2, 3]. However, water has a boiling point of 100 °C, the highest in the series, despite being the lightest group‐16 hydride [4]. A similar trend is seen for group‐17 hydrides, where the boiling points show a V‐shaped dependence on the molecular weight (Figure 1) [4]. The commonly accepted textbook explanation for this anomaly is the presence of exceptionally strong intermolecular interactions, specifically hydrogen bonding, in second‐row hydrides like H_2_O [1, 2, 3, 5]. Classically, hydrogen bonding in water is attributed to the large permanent dipole of the H_2_O molecule and the small size of the hydrogen atom, which enables strong, short‐range Coulomb attractions between partially charged atoms (H–O^δ–^•••^δ+^H–O), thereby increasing its boiling point [1, 2, 3, 5]. While this explanation is intuitive, its quantitative validity remains unclear and warrants closer examination.

**FIGURE 1:**
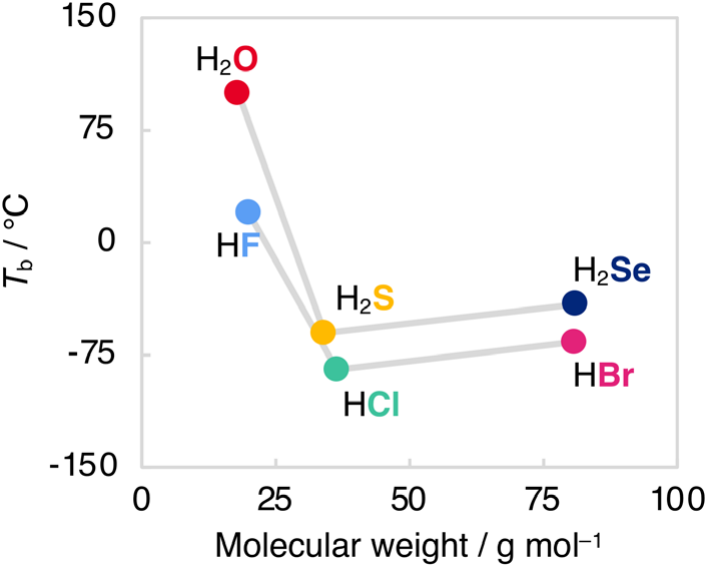
Boiling points versus the molecular weight for representative binary hydrides of elements of groups 16 and 17 of the periodic table [4].

In this work, we quantum chemically investigate the origin of the abnormally high boiling point of water and other small molecules compared to those of their heavier analogs. On the one hand, we confirm that trends in boiling points across binary hydrides of group‐16 elements are related to the strength of two‐body interactions. On the other hand, we show that this strength is *not* determined by the Coulomb attraction between individual monomers. In fact, the relatively strong O•••H–O hydrogen bond in water arises primarily from the shorter intermolecular distance between compact H_2_O molecules rather than from stronger electrostatic forces compared to those of the S•••H–S and Se•••H–Se analogs. This shorter distance, as will become clear throughout the article, is due to significantly reduced steric repulsion, a consequence of the Pauli exclusion principle. The same phenomenon is observed in the binary hydrides of group‐17 elements.

## 2 Results and Discussion

### 2.1 Boiling Points and Intermolecular Interactions

The textbook explanation for trends in the boiling points, *T*_b_, of binary hydrides of groups 16 and 17 elements invokes a simple picture based on two‐body dipole–dipole or van der Waals interactions [1, 2, 3]. In this section, we confirm that this simplified picture is partially correct. Specifically, *T*_b_ trends within a group in the periodic table as a function of molecular weight are dominated by the strength of two‐body interactions.

*T*_b_ and enthalpies of vaporization at *T*_b_, Δ*H*_vap_(*T*_b_), are related through Trouton’s rule, a well‐known empirical relationship stating that the entropy of vaporization at *T*_b_, Δ*S*_vap_(*T*_b_), is approximately constant for many liquids [6, 7, 8].

(1) $$\Delta S_{\mathrm{vap}}(T_{b})=\Delta H_{\mathrm{vap}}(T_{b})/T_{b}$$

For the series of binary hydrides of groups 16 and 17 elements, variations in Δ*S*_vap_(*T*_b_) are smaller than 0.01 kcal K^–1^ mol^–1^ (see Table S1). Consequently, Δ*H*_vap_(*T*_b_) is expected to scale linearly with *T*_b_ through a constant *C*, which corresponds approximately to 1/Δ*S*_vap_(*T*_b_):

(2) $$T_{b}=C\Delta H_{\mathrm{vap}}(T_{b})$$

We graphically illustrate the expected linear relationship between *T*_b_ and the experimental Δ*H*_vap_(*T*_b_) for a series of binary hydrides of groups 16 and 17 elements in Figure 2. Here, we show that *T*_b_ ∝ Δ*H*_vap_(*T*_b_) for this series of compounds, which are commonly used to demonstrate the increase in *T*_b_ with respect to the molecular weight, as well as the anomalies associated with second‐period elements. For example, *T*_b_ increases from −67 and −41 °C for the heavier HBr and H_2_Se to 20 and 100 °C for the lighter HF and H_2_O, while Δ*H*_vap_(*T*_b_) varies from 4.2 and 4.7 to 7.3 and 9.7 kcal mol^–1^, along the same series [9, 10, 11, 12]. Importantly, this relationship also shows that the elevated *T*_b_ for lighter binary hydrides of groups 16 and 17 elements is not caused by entropic effects.

**FIGURE 2:**
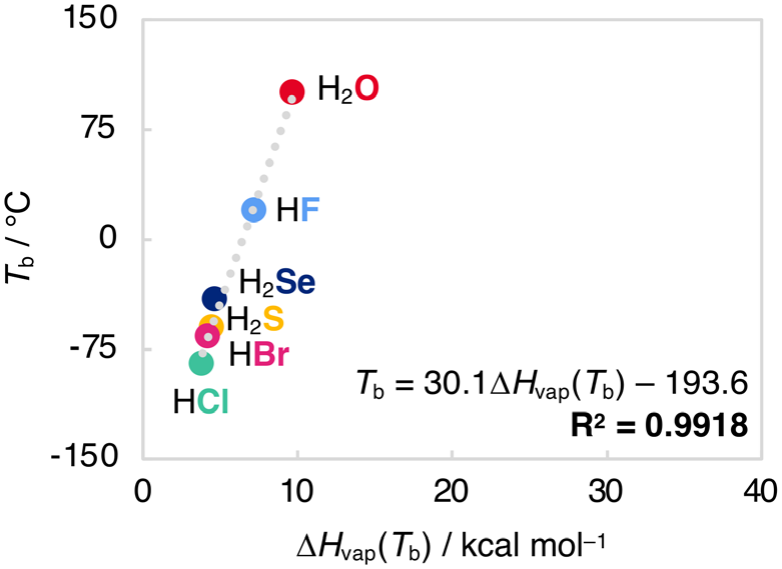
Linear relationship between experimental boiling points and enthalpies of vaporization for representative binary hydrides of groups 16 and 17 elements of the periodic table [4, 9, 10, 11, 12].

Δ*H*_vap_(*T*_b_) is a commonly used proxy to estimate the average strength of the intermolecular interactions of a compound in the liquid phase [6, 7, 8]. Assuming ideal gas behavior, Δ*H*_vap_(*T*_b_) can be approximated as follows:

(3) $$\Delta H_{\mathrm{vap}}(T_{b})\approx –\Delta E_{\mathrm{liq}}+\Delta E_{\mathrm{thermal}}(T_{b})+\Delta nRT_{b}$$

where –Δ*E*_liq_ is the electronic dissociation energy of molecules in the liquid phase, Δ*E*_thermal_(*T*_b_) represents the thermal corrections to the electronic energy at *T*_b_, and Δ*n* *R* *T*_b_ is the work term equal to pV (see Section S1.4). In other words, Δ*H*_vap_(*T*_b_) is directly related to –Δ*E*_liq_, which, in principle, contains pairwise interactions and many‐body effects, and, therefore, *T*_b_ ∝ Δ*H*_vap_(*T*_b_) ∝ –Δ*E*_liq_. Here, we demonstrate that the relationship can be further simplified to two‐body, hydrogen‐bonded dimer dissociation energies, –Δ*E*_dim_, in order to explain the elevated *T*_b_ of HF and H_2_O, provided that the series of compounds shares a similar three‐dimensional structure.

To demonstrate the linear relationship between Δ*H*_vap_(*T*_b_) and –Δ*E*_liq_, we calculate the dissociation energies for *n*‐body [H_2_Ch]_*n*_ and [H_2_X]_*n*_ model clusters (*n* = 3, 4; Ch = O, S, Se; X = F, Cl, and Br), namely, the trimer (–Δ*E*_trim_) and tetramer (–Δ*E*_tetram_) dissociation energies. Although the liquid phase consists of a dynamic ensemble of structures, we employ a systematic set of model clusters that enables investigation of hydrogen bond formation and assessment of the textbook explanation for the observed *T*_b_ trends. To this end, we selected structures that resemble the most common arrangements in the liquid phase while maintaining similar bonding motifs across each [H_2_Ch]_*n*_ or [HX]_*n*_ series. Binary hydrides of groups 16 and 17 elements are known to adopt different three‐dimensional structures in the liquid phase. For instance, H_2_O and H_2_S form branched, cyclic, and three‐dimensional networks [13, 14, 15], whereas HF and HCl are known to form zigzag linear chain structures, as shown by Monte Carlo and molecular dynamics simulations [16, 17, 18]. Our model clusters are constructed to reflect these structural motifs; as such, [H_2_Ch]_*n*_ correspond to true local minima, whereas *C*_s_ constraints were imposed on [HX]_*n*_ to enforce representative structural motifs consistent with known liquid‐phase arrangements (see Figure 3). The ability of these clusters to capture liquid‐phase interactions is supported by the strong correlation between their calculated stabilities and the corresponding experimental thermodynamic data, as discussed below.

**FIGURE 3:**
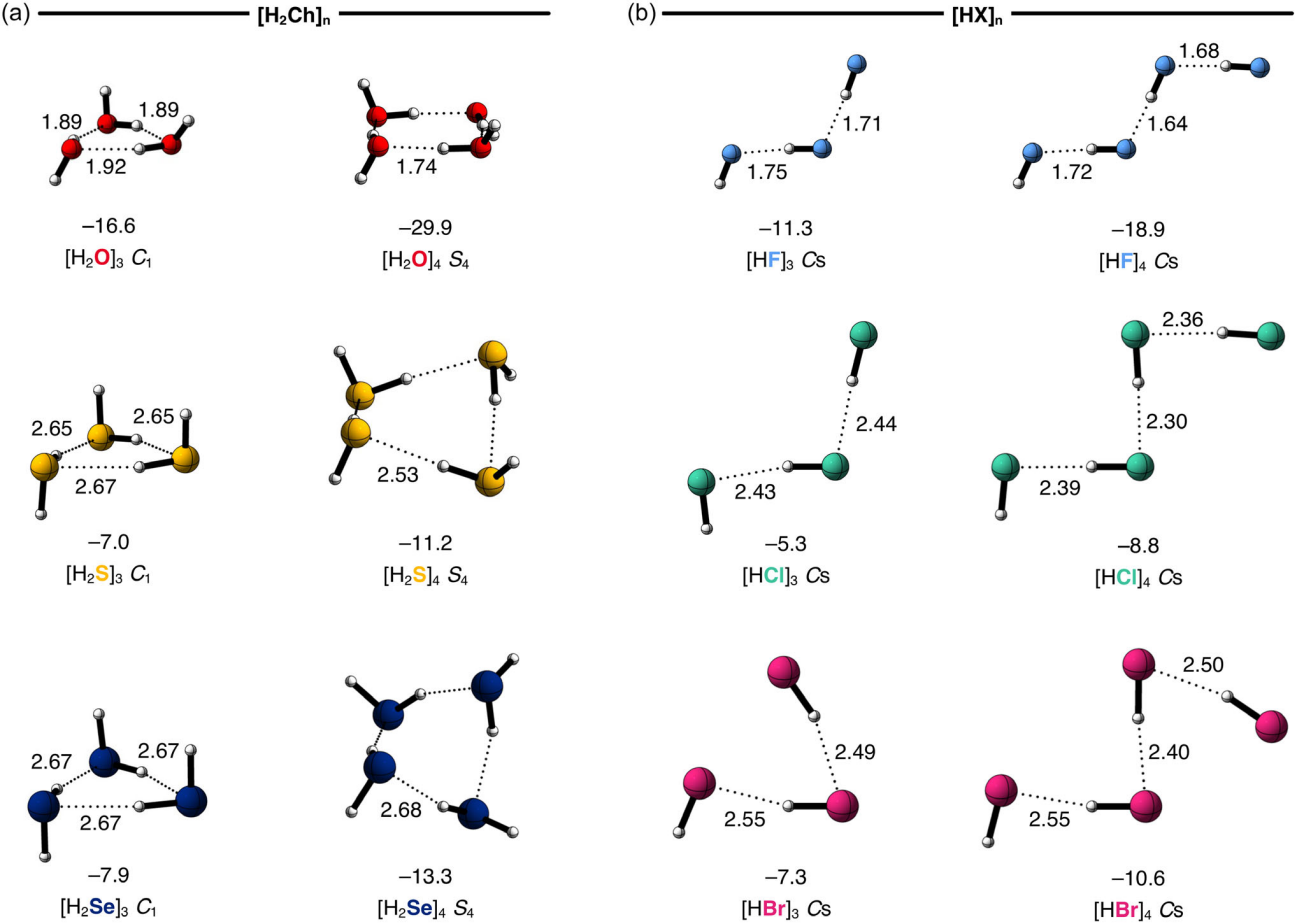
Geometries (in Å) and trimerization and tetramerization energies (in kcal mol^–1^) for model [H_2_Ch]_4_ and [H_2_X]_4_ clusters (Ch = O, S, Se; X = F, Cl, Br). Computed at ZORA‐BLYP‐D3(BJ)/TZ2P.

We find that there is a strong linear relationship between the experimental Δ*H*_vap_(*T*_b_) and both –Δ*E*_trim_ and –Δ*E*_tetram_, showing that our model clusters are representative of the liquid‐phase structures to reproduce the experimental trends of thermodynamic data (Figure 4a). Accordingly, *T*_b_ scales with –Δ*E*_trim_ and –Δ*E*_tetram_ and, thus, *T*_b_ ∝ –Δ*E*_trim_ and *T*_b_ ∝ –Δ*E*_tetram_ (Figure 4b). This leads to our second key conclusion: variations in *T*_b_ across binary hydrides of groups 16 and 17 elements, and thus the elevated *T*_b_ for HF and H_2_O, are dominated by electronic effects, with thermal and enthalpic contributions playing a minor role.

**FIGURE 4:**
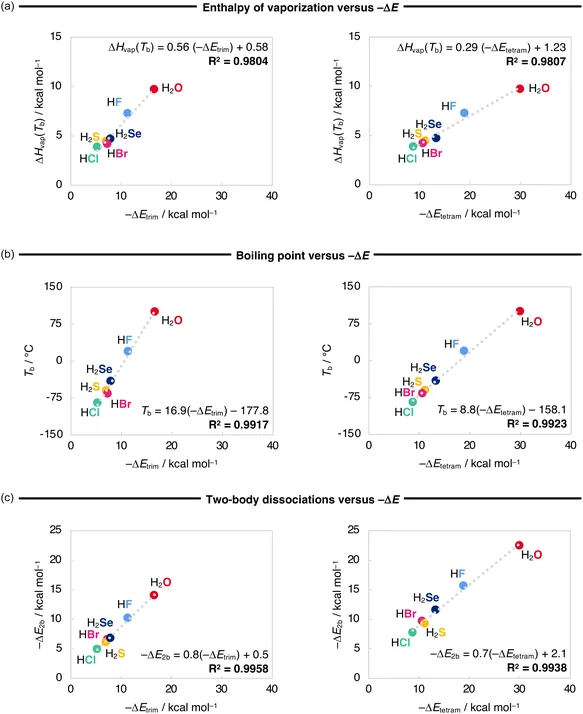
Linear relationship of trimer and tetramer dissociation energies with experimental (a) enthalpies of vaporization, (b) boiling points, and (c) the two‐body term, for representative binary hydrides of groups 16 and 17 elements of the periodic table [4, 9, 10, 11, 12]. Computed at ZORA‐BLYP‐D3(BJ)/TZ2P.

Generally, –Δ*E*_trim_ and –Δ*E*_tetram_ can be written in terms of many‐body expansion terms [19]:

(4a) $$\text{–}\Delta E_{\text{trim}}=\text{–}\Sigma_{i}\Delta E_{\mathrm{strain},i}\;\text{–}\Delta E_{2b}\;\text{–}\Delta E_{3b}$$

(4b) $$\text{–}\Delta E_{\text{tetram}}=\text{–}\Sigma_{i}\Delta E_{\mathrm{strain},i}\;\text{–}\Delta E_{2b}\;\text{–}\Delta E_{3b}\;\text{–}\Delta E_{4b}$$

where Δ*E*_strain,*i*_ is the strain energy of each monomer, the two‐body term, Δ*E*_2b_, represents all pairwise interactions in the cluster, and the three‐ and four‐body terms, Δ*E*_3b_ and Δ*E*_4b_, account for cooperative effects (see the Section S1.5).

In both –Δ*E*_trim_ and –Δ*E*_tetram_, –Δ*E*_2b_ is the leading term and accounts for 75%–93% of the total dissociation energies, whereas –Δ*E*_4b_ is negligible (Table 1). This agrees with a previous study showing that the higher‐order terms are small, becoming negligible, especially for heavier elements [20]. Consequently, –Δ*E*_trim_ and –Δ*E*_tetram_ have a strong linear relationship with –Δ*E*_2b_ (Figure 4c), and thus, *T*_b_ ∝ Δ*H*_vap_(*T*_b_) ∝ –Δ*E*_trim/tetram_ ∝ –Δ*E*_2b_. In principle, this is in line with the classical textbook explanation for trends in the *T*_b_ along binary hydrides of groups 16 and 17 elements [1, 2, 3].

**TABLE 1:** Many‐body expansion of the dimer‐, trimer‐, and tetramer dissociation energies (in kcal mol^–1^). [H_2_Ch]_*n*_ and [HX]_*n*_ clusters at their equilibrium geometries (*n* = 2, 3, 4; Ch = O, S, Se; X = F, Cl, Br).^a^

Systems	–Δ*E*	**–Σ**_***i***_ **Δ*E***_**strain,*i***_	**–Δ*E***_**2b**_	**–Δ*E***_**3b**_	**–Δ*E***_**4b**_
[H_2_O]_2_	5.2	−0.1 (–2%)	5.3 (102%)	—	—
[H_2_S]_2_	2.2	0.0 (0%)	2.2 (100%)	—	—
[H_2_Se]_2_	2.4	0.0 (0%)	2.4 (100%)	—	—
[H_2_O]_3_	16.6	−0.6 (–3%)	14.0 (84%)	3.1 (19%)	—
[H_2_S]_3_	7.0	−0.1 (–1%)	6.1 (87%)	1.0 (14%)	—
[H_2_Se]_3_	7.9	−0.1 (–2%)	6.8 (86%)	1.3 (16%)	—
[HF]_3_	11.3	−0.2 (–2%)	10.2 (90%)	1.4 (12%)	—
[HCl]_3_	5.3	−0.1 (–1%)	4.9 (93%)	0.4 (8%)	—
[HBr]_3_	7.3	−0.1 (–2%)	6.6 (91%)	0.8 (11%)	—
[H_2_O]_4_	29.9	−1.7 (–6%)	22.5 (75%)	8.3 (28%)	0.8 (3%)
[H_2_S]_4_	11.2	−0.2 (–2%)	9.3 (83%)	1.9 (17%)	0.2 (2%)
[H_2_Se]_4_	13.3	−0.2 (–1%)	11.6 (87%)	1.9 (14%)	−0.1 (0%)
[HF]_4_	18.9	−0.6 (–3%)	15.6 (83%)	3.7 (19%)	0.1 (1%)
[HCl]_4_	8.8	−0.2 (–2%)	7.7 (88%)	1.2 (14%)	0.0 (0%)
[HBr]_4_	10.6	−0.3 (–2%)	9.7 (91%)	1.3 (12%)	−0.1 (–1%)

a Percentage contributions to dissociation energies in parentheses. Computed at ZORA‐BLYP‐D3(BJ)/TZ2P.

To understand the trends in –Δ*E*_2b_, we analyze the dissociation energy of simple dimers. In contrast to –Δ*E*_2b_, –Δ*E*_dim_ shows a poorer linear relationship with the experimental Δ*H*_vap_(*T*_b_) (*R* ^2^ = 0.8888) and, consequently, *T*_b_ (*R* ^2^ = 0.8517; see Figure S1). This is because –Δ*E*_dim_ cannot accurately reproduce trends in *T*_b_ for systems with similar molecular weights, such as when comparing group‐16 and group‐17 hydrides containing elements from the same period. For example, –Δ*E*_dim_ increases from 2.2 to 2.4 kcal mol^–1^ from H_2_S to HCl, whereas *T*_b_ decreases from −60 to −85 °C (Figure S1 and Table S2). Nonetheless, –Δ*E*_dim_ already shows stronger interactions for HF and H_2_O, −4.8 and −5.2 kcal mol^–1^, respectively, leading to their elevated *T*_b_.

In fact, H_2_Ch compounds exhibit higher *T*_b_ than HX due to their more branched three‐dimensional structures, which enable the formation of more hydrogen bonds than the zigzag linear chains found in HX (see Figure 3) [20]. This structural difference between H_2_Ch and HX has a non‐negligible impact already at the level of the second‐order term in the many‐body expansion, leading to more destabilizing –Δ*E*_2b_ for [H_2_Ch]_*n*_ compared to the [HX]_*n*_ series (*n* = 3, 4). Notably, this structural effect is not captured by –Δ*E*_dim_ alone at the second‐order level.

On the other hand, there is a strong linear relationship between *T*_b_ and the experimental Δ*H*_vap_(*T*_b_) with the quantum chemically calculated –Δ*E*_dim_ for a series of compounds with the same three‐dimensional structure, e.g., within the series of H_2_O, H_2_S, and H_2_Se or the series HF, HCl, and HBr (Figure 5). These correspond to the standard textbook series in which the abnormally high *T*_b_ of the second‐period elements is observed [1, 2, 3]. Consequently, trends in –Δ*E*_dim_ as a function of molecular weight for H_2_Ch and HX compounds closely mirror those in Δ*H*_vap_(*T*_b_) and, thus, *T*_b_. For example, –Δ*E*_dim_ increases from 2.2 to 2.4 to 5.2 kcal mol^–1^ along [H_2_S]_2_, [H_2_Se]_2_, and [H_2_O]_2_, while *T*_b_ increases from −60 to −41 to 100 °C along the same series (Figure 5b; see also Table S2).

**FIGURE 5:**
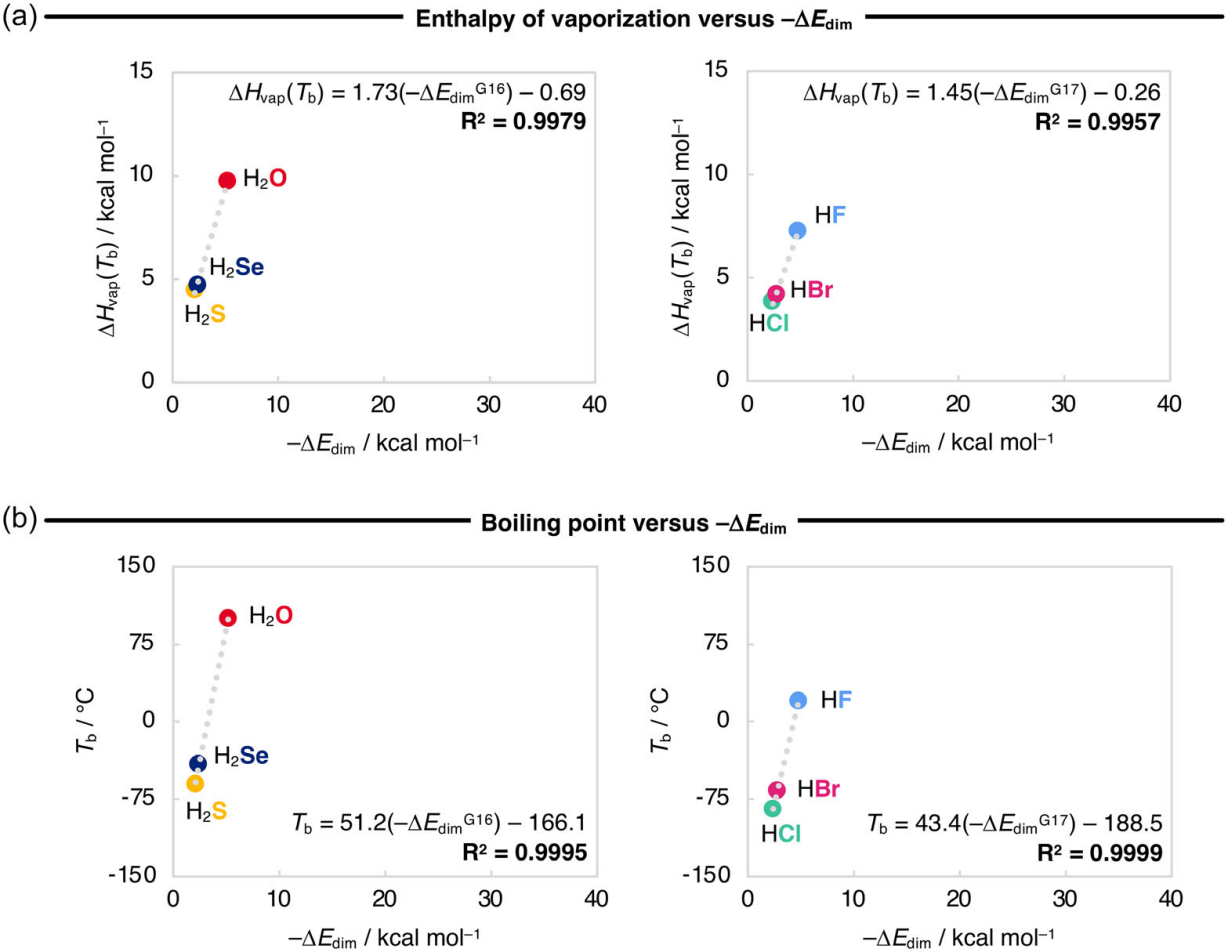
Linear relationship between dimer dissociation energies and experimental (a) enthalpies of vaporization and (b) boiling points for representative binary hydrides of groups 16 and 17 elements of the periodic table [4, 9, 10, 11, 12]. Computed at ZORA‐BLYP‐D3(BJ)/TZ2P.

Therefore, within the series of H_2_Ch and HX, we find that variations in *T*_b_ are largely governed by two‐body interactions that can be approximately reduced to pairwise hydrogen‐bond interactions, i.e., *T*_b_ ∝ Δ*H*_vap_(*T*_b_) ∝ –Δ*E*_dim_. Consistent with this interpretation, a recent work by Hoffman and Xantheas [20] shows that two‐body interactions account for more than 80% of the bond energy in H_2_Ch tetramers. This also explains why pairwise potentials can predict vapor pressures and boiling points of water with surprisingly good accuracy [21, 22, 23, 24].

We emphasize that boiling points are macroscopic properties that depend on a balance of many‐body interactions and entropic effects in the liquid phase. The present cluster‐based analysis does not aim to provide a complete quantitative description of the liquid state or predict the *T*_b_ value of the studied compounds. Rather, it is used to isolate and rationalize the *trends* in intermolecular interaction strengths within chemically similar series, that is, the stronger cohesive forces for the smaller second‐period hydride compounds that lead to their larger Δ*H*_vap_(*T*_b_) and *T*_b_ compared to those of their heavier analogs. The observed correlations between cluster interaction energies and experimental thermodynamic quantities should not be used to predict general trends in *T*_b_. Instead, they suggest that the underlying two‐body hydrogen bond interactions provide a meaningful descriptor of these trends, while entropic, higher‐order many‐body terms and quantum nuclear effects primarily modulate their absolute magnitude.

In summary, we demonstrate that the anomalously high boiling points of second‐period binary hydrides relative to their heavier analogs originate mainly from the bonding mechanism governing their dimerization. This conclusion holds because, within the H_2_Ch or HX series of compounds, the following conditions are satisfied: (i) Δ*S*_vap_(*T*_b_) does not vary significantly [6, 7, 8]; (ii) variations in thermal corrections to the dissociation energies are negligible; and (iii) a roughly similar three‐dimensional structural configuration is adopted in the liquid phase. In the following sections, we investigate the stability of the studied dimers to elucidate the origin of the trends in *T*_b_.

### 2.2 Classical Interpretation of Hydrogen Bonding

Within the series of binary hydrides of group‐16 elements, the H_2_O dimer is significantly more stable than its heavier analogs. In textbooks, this is commonly attributed to water’s well‐known ability to form hydrogen bonds, whereas H_2_S and H_2_Se are generally, although incorrectly, considered incapable of doing so [1, 2, 3]. The classical interpretation of hydrogen bonding suggests that these interactions are primarily Coulombic in nature. According to this view, the larger partial charges on oxygen and hydrogen atoms would lead to stronger electrostatic stabilization in H_2_O compared to that in its heavier analogs [1, 2, 3, 5]. This expectation is supported by an analysis of the molecular electrostatic potentials (MEPs) of the H_2_Ch fragments, which reveal much greater charge separation in H_2_O. For instance, the MEP of H_2_O ranges from −31 to +43 kcal mol^–1^, whereas the range is only −16 to +24 kcal mol^–1^ for H_2_S and −14 to +19 kcal mol^–1^ for H_2_Se (Figure 6b). Similar observations can be made for binary hydrides of group‐17 elements, as reported in Figure S2.

**FIGURE 6:**
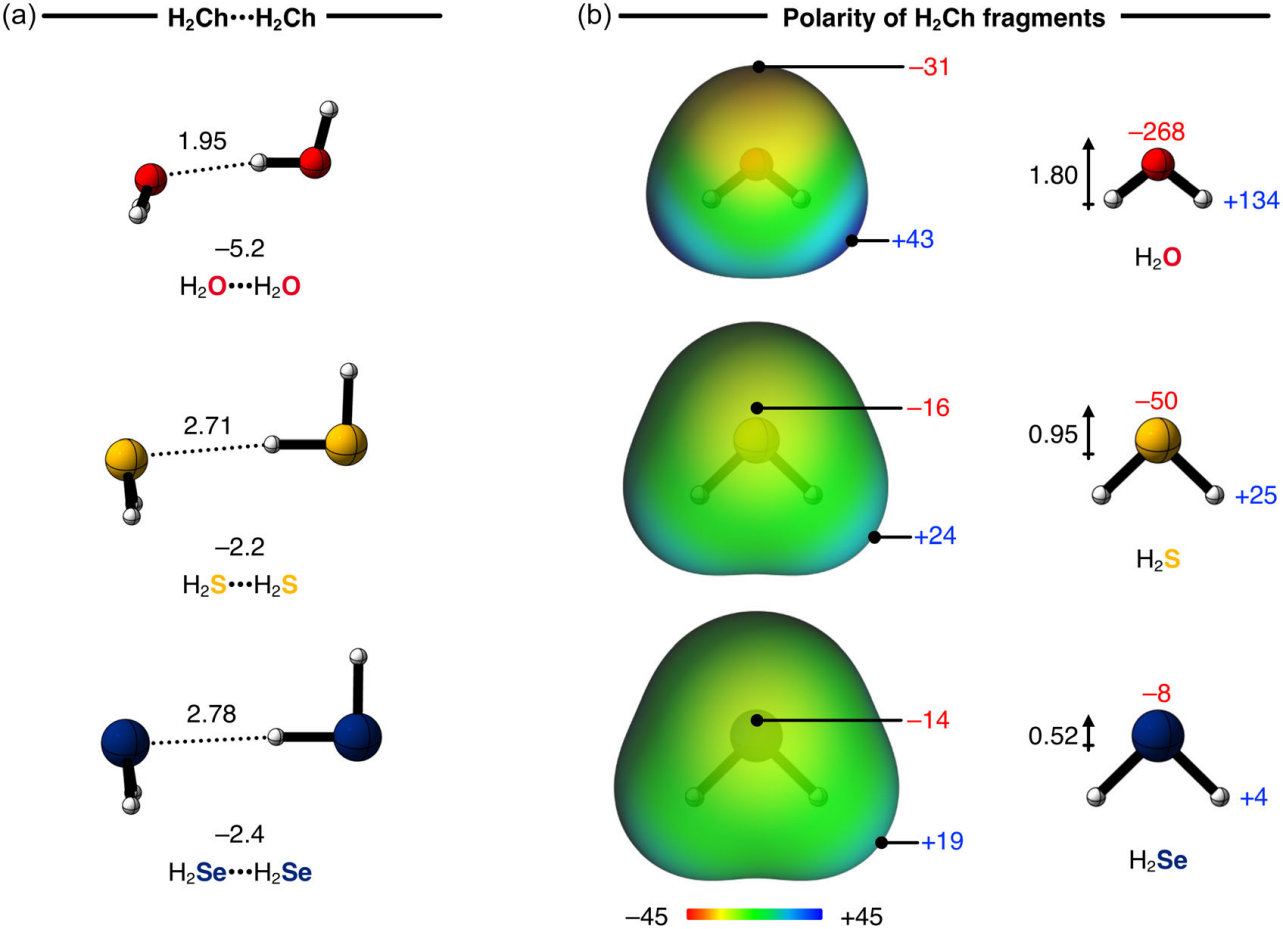
Classical explanation for the abnormal boiling point of water. (a) Equilibrium geometries (in Å) and dimerization energies (in kcal mol^–1^) for H_2_Ch dimers (Ch = O, S, Se), computed at ZORA‐BLYP‐D3(BJ)/TZ2P. (b) Molecular electrostatic potentials (in kcal mol^–1^; isosurfaces at 0.001), dipole moments (in Debye), and Voronoi deformation density charges (in milli‐electrons) of H_2_Ch fragments (Ch = O, S, Se), computed at ZORA‐BLYP‐D3(BJ)/TZ2P.

However, is the stronger Coulombic attraction between H_2_O fragments truly responsible for the high stability of the H_2_O dimer and, consequently, the elevated boiling point of water?

### 2.3 The Exact Coulombic Interactions

We begin by investigating the formation of H_2_Ch•••H_2_Ch (Ch = O, S, Se), starting from the individual H_2_Ch fragments, and plotting their Δ*E* as a function of *r*_Ch•••H_ (Figure 7a). At longer *r*_Ch•••H_ values, all H_2_Ch•••H_2_Ch exhibit comparable stability. However, the energy minima are reached at larger distances for the heavier H_2_S•••H_2_S and H_2_Se•••H_2_Se. In contrast, the formation of H_2_O•••H_2_O proceeds further at shorter *r*_Ch•••H_ values, where its Δ*E* becomes significantly more stabilizing than that of its heavier analogs.

**FIGURE 7:**
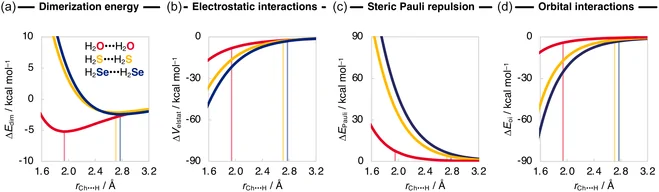
Energy decomposition analysis of the hydrogen‐bonded H_2_Ch•••H_2_Ch dimers (Ch = O, S, Se) as a function of the Ch•••H distance. (a) Total dimerization energies (in kcal mol^–1^). (b) Electrostatic interaction energies between the charge distributions of the H_2_Ch fragments. (c) Destabilizing occupied–occupied orbital interaction energy due to the Pauli exclusion principle. (d) Stabilizing donor–acceptor interaction energies responsible for the covalent component of the hydrogen bond. The vertical lines indicate the equilibrium geometries, and their values are reported in Table 2. Computed at ZORA‐BLYP‐D3(BJ)/TZ2P.

Next, we apply the activation strain model (ASM), which divides Δ*E*_dim_ into two components [25]. The first term is the strain energy (Δ*E*_strain_) associated with the geometrical deformation of the individual fragments as dimerization occurs. For the analyzed H_2_O, H_2_S, and H_2_Se dimers, Δ*E*_strain_ is negligible, amounting to less than 0.1 kcal mol^–1^ (Table 2). The second component is the interaction energy (Δ*E*_int_) between the deformed fragments, which is primarily responsible for the formation of H_2_Ch•••H_2_Ch. In the framework of Kohn–Sham molecular orbital (KS‐MO) theory, Δ*E*_int_ can be further dissected into physically meaningful steps. These steps begin with the wavefunctions of the deformed H_2_Ch fragments and culminate in the final wavefunction of the corresponding H_2_Ch dimer. The energy associated with each step is quantified using our energy decomposition analysis (EDA) method, which has been explained in detail elsewhere [25]. The contribution of weak dispersion forces to the Δ*E*_int_ profile and, thus, to the trends in boiling points is negligible, as the dispersion energy curves (Δ*E*_disp_) are nearly constant and, therefore, are not discussed here (see Figures S3 and S4).

**TABLE 2:** Activation strain and energy decomposition analyses of H_2_Ch•••H_2_Ch dimers a consistent *r*_Ch•••H_ and at the equilibrium geometries (Ch = O, S, Se). Bond lengths in Å and energies in kcal mol^–1^. Computed at ZORA‐BLYP‐D3(BJ)/TZ2P.

Dimers	***r***_**Ch•••H**_	**Δ*E***_**dim**_	**Δ*E***_**strain**_	**Δ*E***_**int**_	**Δ*V***_**elstat**_	**Δ*E***_**Pauli**_	**Δ*E***_**oi**_	**Δ*E***_**disp**_
Consistent geometries								
H_2_O•••H_2_O	2.800	−2.61	0.00	−2.61	−2.17	0.53	−0.60	−0.37
H_2_S•••H_2_S	2.800	−2.11	0.01	−2.12	−2.38	3.40	−1.77	−1.37
H_2_Se•••H_2_Se	2.800	−2.41	0.01	−2.42	−2.81	5.20	−2.95	−1.86
Equilibrium geometries^a^								
H_2_O•••H_2_O	1.947	−5.20	0.05	−5.25	−8.02	7.64	−4.08	−0.78
H_2_S•••H_2_S	2.711	−2.15	0.02	−2.17	−2.84	4.37	−2.20	−1.50
H_2_Se•••H_2_Se	2.775	−2.43	0.02	−2.45	−3.04	5.58	−3.13	−1.86

a Different decomposition schemes lead to similar results for analyses at the equilibrium gemetries [26, 27, 28, 29].

In the first step, we quantify the electrostatic interactions (Δ*V*_elstat_) between the unperturbed charge distributions of the H_2_Ch fragments during dimer formation (Figure 7b). Our quantum chemical calculations reveal that the H_2_O dimer experiences the *least* stabilizing electrostatic attraction within the H_2_Ch dimer series. Specifically, the Δ*V*_elstat_ curves become progressively more stabilizing along the series H_2_O•••H_2_O, H_2_S•••H_2_S, and H_2_Se•••H_2_Se (Figure 7b). Thus, the Δ*V*_elstat_ curve for H_2_O•••H_2_O is the least stabilizing in the case that two H_2_O molecules approach each other from an infinite separation.

This occurs despite H_2_O being the most polar fragment with the largest charge separation. This apparent contradiction arises because atoms and molecules do not behave as point charges but rather as dispersed charge distributions[30]. In reality, the larger positively charged nuclei and more diffuse negatively charged electron densities of the heavier H_2_S and H_2_Se fragments allow for a greater overlap of their charge distributions, resulting in more stabilizing electrostatic attraction in their corresponding dimers compared to H_2_O. Therefore, Coulombic interactions are not directly responsible for the unexpectedly high boiling point of water. Instead, as will become clear in the next sections, they would predict a monotonic increase in *T*_b_ along H_2_O•••H_2_O, H_2_S•••H_2_S, and H_2_Se•••H_2_Se.

The misleading assumption that more polar H_2_O should exhibit more stabilizing Δ*V*_elstat_ than its heavier analogs arises from differences in the intermolecular Ch•••H distances (*r*_Ch•••H_) rather than from the dipole moments of the H_2_Ch monomers. As noted above, Coulombic interactions arise from the distance‐dependent overlap of charge distributions. At longer *r*_Ch•••H_, Δ*V*_elstat_ is more stabilizing for H_2_S•••H_2_S and H_2_Se•••H_2_Se due to their more diffuse charged distributions (Figure 7b). For example, at 2.80 Å, Δ*V*_elstat_ varies from −2.2 to −2.4 to −2.8 kcal mol^–1^ across H_2_O•••H_2_O, H_2_S•••H_2_S, and H_2_Se•••H_2_Se (Table 2).

However, H_2_S•••H_2_S and H_2_Se•••H_2_Se reach their equilibrium distances earlier along *r*_Ch•••H_, at 2.71 Å and 2.78 Å, respectively, whereas the equilibrium geometry of H_2_O•••H_2_O occurs at a significantly shorter *r*_Ch•••H_ of 1.95 Å. This shorter equilibrium distance allows a greater overlap of charge distributions in H_2_O•••H_2_O, resulting in a more stabilizing Δ*V*_elstat_ compared with H_2_S•••H_2_S and H_2_Se•••H_2_Se. For example, at the equilibrium geometries, Δ*V*_elstat_ varies from −8.0 to −2.8 to −3.0 kcal mol^–1^ across H_2_O•••H_2_O, H_2_S•••H_2_S, and H_2_Se•••H_2_Se (Table 2).

In summary, water experiences more attractive Coulomb interactions because of the shorter intermolecular O•••H distance, not because of its larger dipole moment. The same conclusion applies to the HX•••HX series (X = F, Cl, and Br; see Table S2). This raises the question: why can H_2_O form shorter Ch•••H bonds than its heavier analogs?

### 2.4 The Origin of the Abnormally High Boiling Point of Water

In the next step of our analysis, a fundamental quantum mechanical condition must be satisfied: two electrons in the same system cannot occupy the same spin orbital, i.e., they cannot have identical sets of quantum numbers [31]. In other words, electrons in two different spin orbitals cannot be simultaneously at the same point in space. This is the Pauli exclusion principle, which is manifested when there is overlap between same‐spin‐occupied orbitals. The Pauli principle is satisfied by antisymmetrizing the product wavefunction of the two H_2_Ch fragments, which ensures the correct fermionic nature of the total wavefunction. The associated energy increase due to the required orthogonalization of the occupied orbitals is referred to as the steric Pauli repulsion (Δ*E*_Pauli_).

Our analysis shows that the unexpectedly high boiling point of water arises from the more compact lone‐pair (LP) orbitals on the oxygen atom of the H_2_O fragment, which introduce significantly less Δ*E*_Pauli_ compared to the more diffuse LP orbitals of H_2_S and H_2_Se during dimer formation. As shown in Figure 7c, the Δ*E*_Pauli_ curves become progressively less destabilizing along the series H_2_Se•••H_2_Se, H_2_S•••H_2_S, and H_2_O•••H_2_O (see numerical examples at consistent geometries in Table 2). This trend occurs because Δ*E*_Pauli_ is proportional to the overlap squared of same‐spin‐occupied orbitals [25]. As the LP_Ch_ orbitals become more compact from H_2_Se to H_2_S to H_2_O, the destabilizing occupied–occupied orbital overlap squared between the LP_Ch_ and the σ_H__─__Ch_ bonding orbital (σ_H–Ch_) on the opposite H_2_Ch fragment (*S* ^2^_LP–σ_) decreases, leading to a reduction in Δ*E*_Pauli_ across the series (Figure 8).

**FIGURE 8:**
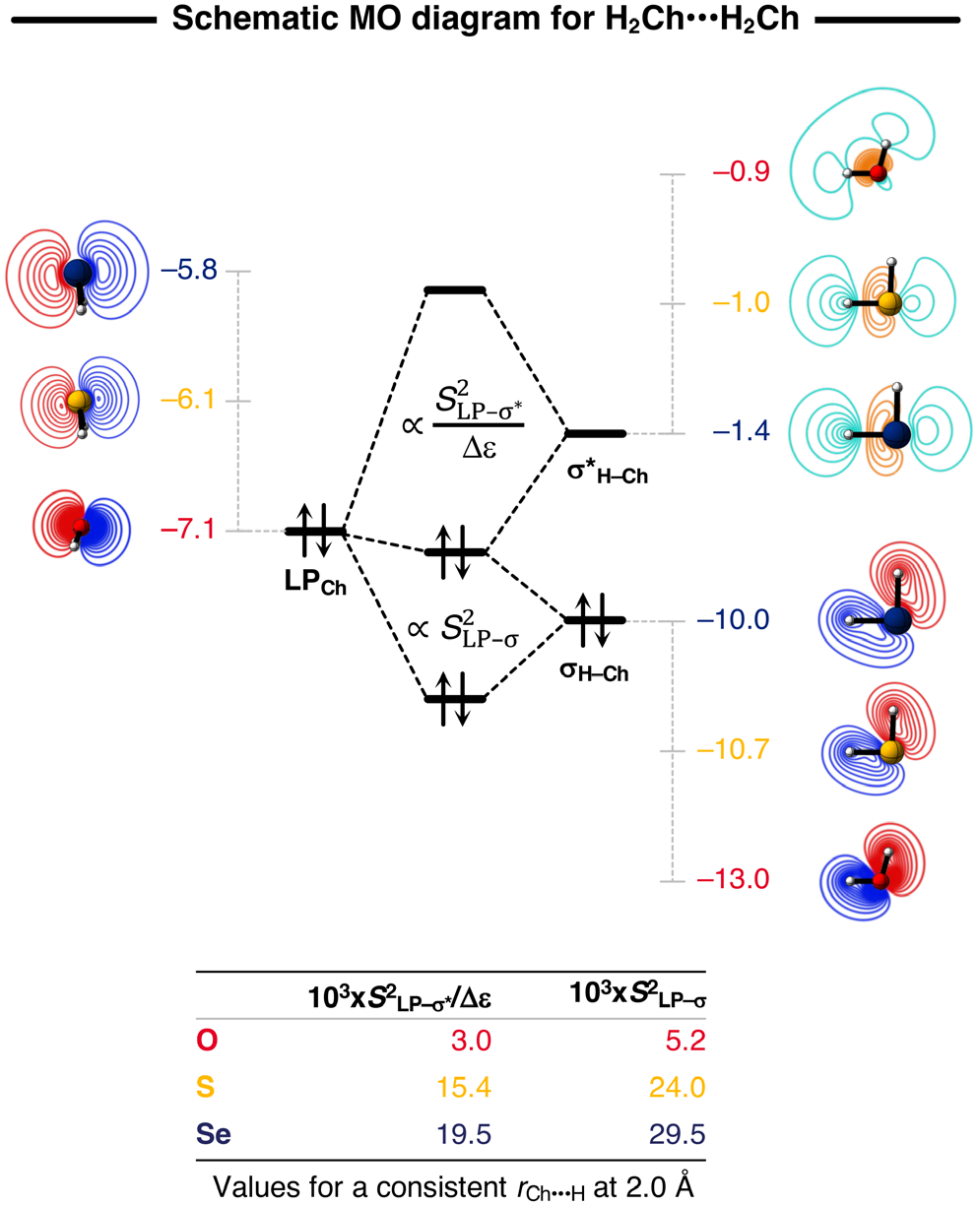
Schematic molecular orbital diagram for the hydrogen‐bonded H_2_Ch•••H_2_Ch dimers (Ch = O, S, Se). Orbital energies (in eV) and orbital overlaps for a consistent *r*_Ch•••H_ at 2.0 Å. Values at equilibrium geometries are reported in Table S3. Computed at ZORA‐BLYP‐D3(BJ)/TZ2P.

Crucially, Δ*E*_Pauli_ is the only term acting as a repulsive wall, preventing the two H_2_Ch fragments from approaching each other until they reach the equilibrium geometry of their respective H_2_Ch dimers (Figure 7c). Δ*V*_elstat_ and Δ*E*_oi_ remain essential stabilizing components. However, they do not determine the trends in Δ*E*_dim_ as their magnitude is strongly dependent on the intermolecular distance set by Δ*E*_Pauli_. In other words, Δ*E*_Pauli_ emerges as the dominant factor controlling the equilibrium distance and, thereby, indirectly governs the strength of all stabilizing interactions. Due to the reduced Δ*E*_Pauli_, H_2_O•••H_2_O can achieve a shorter *r*_Ch•••H_, where all stabilizing interactions, including Δ*V*_elstat_ and Δ*E*_oi_, become more favorable. The latter is discussed in the following section. The same conclusions are drawn for dimers of binary hydrides of group‐17 elements, as can be seen in Figure S4, which shows that the Δ*E*_Pauli_ curve is significantly less destabilizing for HF•••HF, whereas Δ*V*_elstat_ and Δ*E*_oi_ become more favorable for HCl•••HCl and HBr•••HBr.

To demonstrate this, we calculate Δ*E*_dim_(*r*_Ch•••H_) for fictitious H_2_Ch dimers, *fict*‐[H_2_Ch•••H_2_Ch], which are forced to experience the same Δ*E*_Pauli_(*r*_Ch•••H_) as H_2_O•••H_2_O (Figure 9a). In this scenario, steric repulsion is enforced to have no impact on the trends in Δ*E*_dim_, which are, then, fully governed by Coulomb attractions and donor–acceptor interactions. Notably, *fict*‐[H_2_O•••H_2_O] ≡ H_2_O•••H_2_O retains the same Δ*E*_dim_ curve as shown in Figure 7a, albeit on a different scale in Figure 9a.

**FIGURE 9:**
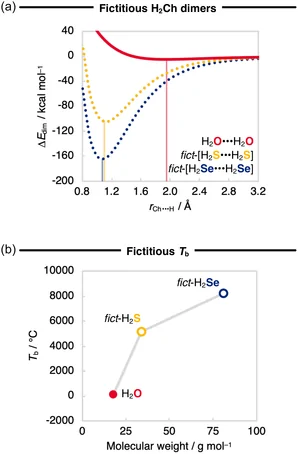
(a) Dimerization energy for the fictitious H_2_Ch dimers (Ch = O, S, Se), which are forced to experience the same Δ*E*_Pauli_(*r*_Ch•••H_) as H_2_O•••H_2_O, and (b) the associated fictitious boiling points obtained using the linear relationship in Figure 5b. Computed at ZORA‐BLYP‐D3(BJ)/TZ2P.

In contrast, the reduced Δ*E*_Pauli_ in *fict*‐[H_2_S•••H_2_S] and *fict‐*[H_2_Se•••H_2_Se] leads to significantly shorter equilibrium distances and more strongly bound dimers (Figure 9a). As shown previously, Δ*V*_elstat_(*r*_Ch•••H_) and Δ*E*_oi_(*r*_Ch•••H_) become increasingly favorable as Ch increases in size (Figure 7b,d). Consequently, Δ*E*_dim_(*r*_Ch•••H_) becomes progressively more stabilizing along the series H_2_O•••H_2_O, *fict*‐[H_2_S•••H_2_S], and *fict*‐[H_2_Se•••H_2_Se]. In addition, Δ*E*_Pauli_(*r*_Ch•••H_) is not enough to prevent the close approach of the heavier H_2_Ch fragments, and the Δ*E*_dim_ minima shift from approximately 2.75 Å in [H_2_S•••H_2_S] and [H_2_Se•••H_2_Se] to approximately 1.10 Å in *fict*‐[H_2_S•••H_2_S] and *fict*‐[H_2_Se•••H_2_Se].

Without the increased Δ*E*_Pauli_ as Ch increases in size along the H_2_Ch dimers, the anomaly in *T*_b_ trends across binary hydrides of group‐16 elements would not exist. We emphasize that the linear relationship between *T*_b_ and –Δ*E*_dim_ in Figure 5b cannot be used to predict general trends in *T*_b_. However, as a mere thought experiment, we used it to qualitatively estimate the *T*_b_ for *fict*‐H_2_S and *fict*‐H_2_Se. Accordingly, *T*_b_ is expected to significantly increase along with H_2_O, *fict*‐H_2_S, and *fict*‐H_2_Se (Figure 9b). In reality, such extremely high *T*_b_ values are not observed for H_2_S and H_2_Se because they cannot form short‐range pairwise interactions in the liquid phase. See Figure S6 for similar analyses of fictitious dimers of binary hydrides of group‐17 elements.

In summary, the anomalous high boiling points for the second‐period binary hydrides of groups 16 and 17 arise from their compact nature, which enables shorter and more strongly bound pairwise interactions. Although these compact molecules also happen to possess larger dipole moments, the dipole moment itself is not the physical origin of their stronger pairwise interactions.

### 2.5 The Quantum Chemical Interpretation of Hydrogen Bonding

In the final step, mixing occurs between the occupied and unoccupied orbitals of the two H_2_Ch fragments. This leads to orbital relaxation in the antisymmetric wavefunction of H_2_Ch•••H_2_Ch, i.e., H_2_Ch dimers are stabilized, yielding the final wavefunction of H_2_Ch•••H_2_Ch. The energy change associated with this process is referred to as the orbital interaction energy (Δ*E*_oi_) [25].

Hydrogen bonds are not purely electrostatic in nature; they also exhibit an important covalent component arising from donor–acceptor interactions [32, 33, 34, 35, 36, 37]. In H_2_Ch dimers, these donor–acceptor interactions occur when the LP_Ch_ orbital on one fragment donates charge into the empty σ∗ H–Ch antibonding orbital (σ∗_H–Ch_) on the opposite fragment (Figure 8).

This stabilizing interaction is not unique to water. In fact, the covalent contribution to the H_2_Ch•••H_2_Ch hydrogen bond becomes increasingly stabilizing as the group‐16 element becomes heavier. As shown in Figure 7d, the Δ*E*_oi_ curves become more stabilizing from H_2_O•••H_2_O to H_2_S•••H_2_S to H_2_Se•••H_2_Se (see numerical examples at consistent geometries in Table 2). This trend arises because Δ*E*_oi_ is proportional to the orbital overlap squared between LP_Ch_ and σ∗_H–Ch_ (*S* ^2^_LP–σ*_) and inversely proportional to their orbital energy gap (Δε). Heavier group‐16 elements exhibit more diffuse LP_Ch_ orbitals and lower‐energy σ∗_H–Ch_ orbitals, both of which contribute to more stabilizing Δ*E*_oi_ by increasing *S* ^2^_LP–σ*_ and decreasing Δε, respectively (Figure 8). Consequently, the bonding components of the hydrogen bonding (Δ*V*_elstat_ and Δ*E*_oi_) strengthen for heavier group‐16 elements, potentially leading to more stable dimers and higher boiling points for H_2_S•••H_2_S and H_2_Se•••H_2_Se. The same conclusion is found for many(n)‐body systems of groups 16 and 17, that is, the electrostatic and covalent components are more stabilizing and would favor the formation of more compact clusters for heavier (H_2_Ch)_*n*_ and (HX)_*n*_ systems. For example, at an average hydrogen bond length of 2.00 Å, Δ*V*_elstat_ varies from −30.9 to −76.3 kcal mol^–1,^ and Δ*E*_oi_ varies from −18.8 to −109.1 kcal mol^–1^ along the [H_2_O]_4_, [H_2_S]_4_, and [H_2_Se]_4_ series (Table S4). However, this potential for forming stronger hydrogen bonds is counteracted by the increased Pauli repulsion, which imposes a large repulsive wall. Along the same series, Δ*E*_Pauli_ varies from 25.8 to 185.6 kcal mol^–1^ at an average hydrogen bond length of 2.00 Å. Consequently, heavier binary hydrides are forced to adopt longer and weaker intermolecular interactions.

## 3 Conclusion

We provide a quantum‐chemical explanation based on quantitative Kohn–Sham molecular orbital theory for the origin of water’s anomalously high boiling point relative to its heavier analogs in the binary hydrides of group‐16 elements, as described in general chemistry textbooks [1, 2, 3]. Alongside thermodynamic and structural effects, the key lies in the stronger intermolecular interactions experienced by H_2_O molecules in contrast to those of their heavier analogs, such as H_2_S and H_2_Se. However, contrary to the classical explanation often found in textbooks, this is not due to water’s larger dipole moment. In reality, quantum mechanics reveals that molecules do not behave as simple point charges. Despite being less polar, heavier molecules can exhibit stronger, *not weaker*, Coulombic attractions due to a larger overlap between their more diffuse charge distributions.

Water and other small molecules experience significantly less steric Pauli repulsion. This quantum mechanical effect arises from the Pauli exclusion principle, which prevents fermions, e.g., electrons, from occupying the same quantum state. Consequently, when electron‐rich regions of different molecules approach each other, their overlapping spin orbitals force electrons into higher‐energy, unstable states. Small molecules, which contain fewer electrons and more compact orbitals, are less affected by Pauli repulsion. This mechanism contributes to the formation of shorter and stronger intermolecular interactions. In the case of water, the compactness of the H_2_O molecule ultimately contributes to its stronger intermolecular interactions and an unusually high boiling point. While the present analysis is restricted to binary hydrides of groups 16 and 17 elements, the underlying mechanism, namely, the interplay between Pauli repulsion and distance‐dependent stabilization, may be expected to operate more broadly in other hydrogen‐bonded systems, although further investigation is required to assess its generality.

The hydrogen bond is not unique to water, but it is also present in H_2_S and H_2_Se. Contrary to the classical mechanical view, hydrogen bonding has a significant covalent characteristic. Like the Coulombic and electrostatic component, the covalent contribution to hydrogen bonding strengthens as the group‐16 element increases in size. Together, these Coulombic and covalent components could, in principle, lead to elevated boiling points for heavier molecules. However, larger molecules are unable to form shorter and stronger intermolecular interactions due to the increased steric repulsion arising from the quantum mechanical Pauli principle.

## Funding

This work was supported by the Nederlandse Organisatie voor Wetenschappelijk Onderzoek (OCENW.M.21.035).

## Conflicts of Interest

There are no conflicts of interest to declare.

## Supporting information

(Tables S1–S8 and Figures S1–S6) [4, 6, 7, 8, 10, 11, 12, 19, 25, 38, 39, 40, 41, 42, 43, 44, 45, 46, 47, 48, 49, 50, 51, 52, 53, 54, 55, 56, 57].

## Data Availability

The data supporting this article have been included as part of the Supporting Information.
