# Supplementary material for: The Anomalously High Boiling Point of Water Revisited
Source: ChemistryOpen. 2026 Oct 13;15(11):e70316. doi: 10.1002/open.70316 (PMC13661295; doi:10.1002/open.70316)
Supplement: Supplementary file 1 [file OPEN-15-e70316-s001.pdf]

Supporting Information for

The anomalously high boiling point of water revisited

Lucas de Azevedo Santos[1], Siebe Lekanne Deprez[1], F. Matthias Bickelhaupt[1,2,3] and Célia Fonseca Guerra[1*]

[1] Department of Chemistry and Pharmaceutical Sciences, AIMMS, Vrije Universiteit Amsterdam, De Boelelaan 1108, 1081 HZ Amsterdam, The Netherlands.

[2] Institute for Molecules and Materials, Radboud University, Heyendaalseweg 135, 6525 AJ Nijmegen, The Netherlands.

[3] Department of Chemical Sciences, University of Johannesburg, Auckland Park, Johannesburg 2006, South Africa.

* Email: c.fonsecaguerra@vu.nl.

Table of Contents

1. Computational Methods **S2**
1.1. Computational details
1.2. Bond analyses
1.3. Voronoi deformation density (VDD) charge analyses
1.4. Thermochemistry
1.5. Many-body expansion

2. Physicochemical properties of H_2Ch and HX compounds **S6**
Table S1
Figure S1

3. Stability and polarity of HX model dimers **S7**
Figure S2

4. Activation strain and energy decomposition analyses as a function of the bond distance **S8**
Figure S3
Figure S4
Figure S5

5. Activation strain, energy decomposition, and quantitative MO analyses at equilibrium geometries **S11**
Table S2
Table S3

6. Activation strain and energy decomposition analyses at consistent geometries **S12**
Table S4

7. Dimerization energies of fictitious HX dimers **S13**
Figure S6

8. Cartesian coordinates and electronic energies **S14**
Table S5
Table S6
Table S7
Table S8

9. References **S18**

1. Computational Methods

1.1. Computational details

All calculations were carried out using the Amsterdam Modeling Suite (AMS) 2023.101 program.[38-40] All stationary points and energies were obtained using relativistic, dispersion-corrected density functional theory at ZORA-BLYP-D3(BJ)/TZ2P (see Tables S5, S6, S7, and S8 for the Cartesian coordinates). This approach comprises the BLYP level of the generalized gradient approximation (GGA); the exchange functional developed by Becke (B), and the GGA correlation functional developed by Lee, Yang, and Parr (LYP).[41,42] The empirical DFT-D3(BJ) correction developed by Grimme and co-workers,[43-44] which contains the damping function proposed by Becke and Johnson,[45] is used to account for non-local dispersion interactions. Scalar relativistic effects are accounted for using the zeroth-order regular approximation (ZORA).[46] This level has been proven to accurately describe weak interactions and hydrogen bonds.[47-49] Molecular orbitals (MO) were expanded in a large, uncontracted set of Slater-type orbitals (STOs) containing diffuse functions: TZ2P.[50] The basis set is of triple-ζ quality augmented with polarization functions, *i.e.*, one 2p and one 3d set on H; one 3d and one 4f set on O, S, F, and Cl; one 4d and one 4f set on Se and Br. All electrons were included in the variational process, i.e., no frozen core approximation was applied. The accuracies of both the Zlm fitting scheme[51] and the Becke integration grid[52] were set to 'EXCELLENT'. HX trimers and tetramers geometries were computed under C_s symmetry to enforce zigzag linear-chain arrangements.

1.2. Bond analyses

Insight into the bonding mechanism of the $H_2Ch\cdots H_2Ch$ and $HX\cdots HX$ hydrogen bonds (Ch = O, S, Se; X = F, Cl Br) is obtained by using the activation strain model,[25] which is a fragment-based approach to understanding the energy profile associated with a chemical process in terms of the original fragments. The total bond energy, ΔE, profile has been generated by decreasing and increasing the Ch/X•••H bond distances ($r_{Ch/X\cdots H}$) starting from the equilibrium geometries of $H_2Ch\cdots H_2Ch$ and $HX\cdots HX$, while keeping the Ch/X•••H–Ch/X angle frozen. Then, ΔE is decomposed into the strain energy ΔE_{strain}, which is associated with the geometrical deformation of the individual fragments as the process takes place, plus the actual interaction energy ΔE_{int} between the deformed fragments:

$$\Delta E = \Delta E_{strain} + \Delta E_{int} \qquad \text{(S1)}$$

The interaction energy ΔE_{int} between the deformed reactants is further analyzed in the conceptual framework provided by the quantitative Kohn-Sham MO model.[25] To this end, it is decomposed

into physically meaningful terms, using a quantitative energy decomposition analysis (EDA)[25] as a function of $r_{Ch/X\cdots H}$ along the range of 1.6 Å to 3.2 Å:

$$\Delta E_{int}(r_{Ch/X\cdots H}) = \Delta V_{elstat}(r_{Ch/X\cdots H}) + \Delta E_{Pauli}(r_{Ch/X\cdots H}) + \Delta E_{oi}(r_{Ch/X\cdots H}) + \Delta E_{disp}(r_{Ch/X\cdots H}) \quad \text{(S2)}$$

where ΔV_{elstat} is the Coulomb interaction between the unperturbed charge distributions of the deformed reactants which is usually stabilizing and comprises four components: (i) the electron–electron electrostatic repulsion between the electron densities of fragments 1 and 2, $\Delta V_{elstat,\rho_1\rho_2}$; (ii) the nuclei–electron electrostatic attraction between the nuclei of fragment 1 and the electron density of fragment 2, $\Delta V_{elstat,n_1\rho_2}$; (iii) the electron–nuclei electrostatic attraction between the electron density of fragment 1 and the nuclei of fragment 2, $\Delta V_{elstat,\rho_1 n_2}$; and (iv) the nuclei–nuclei electrostatic repulsion between the nuclei of fragments 1 and 2, $\Delta V_{elstat,n_1 n_2}$. The Pauli repulsion energy (ΔE_{Pauli}) comprises the destabilizing interactions between occupied orbitals on either fragment (more precisely, between same-spin occupied spin-orbitals on either fragment) and arises from the antisymmetrization of the Hartree wavefunction due to the Pauli principle. The orbital-interaction energy (ΔE_{oi}) accounts for charge transfer, that is, the interaction between occupied orbitals of one fragment with unoccupied orbitals of the other fragment, including the interactions of the highest occupied and lowest unoccupied MOs (HOMO–LUMO), and polarization, that is, empty–occupied orbital mixing on one fragment, due to the presence of another fragment. ΔE_{disp} accounts for the empirical dispersion corrections as introduced by Grimme et al.[43,44] To facilitate the analyses, the ASM and EDA were performed using the PyFrag 2019 program.[53,54] The qualitative trends obtained from our EDA are insensitive to the choice of density functional.[25] To demonstrate this, we repeated our analyses at the ZORA-M06-2X/TZ2P//ZORA-BLYP-D3(BJ)/TZ2P level of theory[55,56] (see Figure S5).

1.3. Voronoi deformation density (VDD) charge analysis

The Voronoi Deformation Density (VDD) charge analysis allows for the quantification of the flow of electronic charge as a consequence of chemical-bond formation.[57] In the H_2Ch and HX fragments, the VDD atomic charge of an atom A is computed by the spatial integration of the deformation density over the Voronoi cell of atom A, which is the space defined by the bond midplanes on and perpendicular to all bond axes between this atom A and its neighboring atoms:

$$Q_A = -\int_{\text{Voronoi cell of A}} \left[\rho(r) - \textstyle\sum_i \rho_i(r)\right] dr \quad \text{(S3)}$$

where the deformation density $\Delta\rho(\mathrm{r}) = [\rho(r) - \sum_i \rho_i(r)]$ is the density change going from a superposition of the original atomic densities at the positions of the molecule to the actual density of that molecule. This atomic or so-called promolecular density is defined as the sum of the (spherically averaged) ground-state atomic densities $\sum_i \rho_i(r)$. This is the fictitious state in which the charge density has not been affected by chemical bonding and in which all atoms have zero charge. Then, Q_A represents the amount of charge that, due to chemical bonding, flows from a position closer to another nucleus to a position closer to the nucleus of A ($Q_A < 0$), or from a position closer to the nucleus of A to a position closer to another nucleus ($Q_A > 0$).

1.4. Thermochemistry

Enthalpies at a given temperature (T) and 1 atm (H_T) can be calculated from electronic energies (E) and vibrational frequencies using standard thermochemistry relations for an ideal gas for which the work term pV is equal to nRT [eqn (S4)].[6–8]

$$H_T = E + E_{trans,T} + E_{rot,T} + E_{vib,0} + \Delta(E_{vib,0})_T + n\mathrm{RT} \quad \text{(S4)}$$

Here, $E_{trans,T}$, $E_{rot,T}$, and $E_{vib,0}$ are, respectively, the translational, rotational, and zero-point vibrational energy corrections. $\Delta(E_{vib,0})_T$ is the change in the vibrational energy correction when going from 0 K to any T. The vibrational energy corrections are based on our frequency calculations. Together, these contributions are the thermal corrections to the electronic energy, $E_{thermal,T}$ [eqn (S5)). The sum $E + E_{thermal,T}$ is the internal energy, U_T [eqn (S6)].

$$H_T = E + E_{thermal,T} + n\mathrm{RT} \quad \text{(S5)}$$

$$H_T = U_T + n\mathrm{RT} \quad \text{(S6)}$$

Thermal electronic contributions are neglected.

1.5. Many-body expansion

The electronic bond energy of an intermolecular cluster, an *n*-mer, $\Delta E_{n\text{-mer}}$, is generally calculated as

$$\Delta E_{n\text{-mer}} = E_{ijkl...n} - \sum_i^n E_i \tag{S7}$$

Here, $E_{ijkl...n}$ is the electronic energy of the *n*-mer evaluated at its equilibrium geometry, and $\sum_i^n E_i$ is the sum of the monomer electronic energies in their isolated equilibrium geometries. The many-body expansion expresses $\Delta E_{n\text{-mer}}$ in terms of corrections arising from *n*-body interactions ($n > 1$).[19]

$$\Delta E_{n\text{-mer}} = \Delta E_{1b} + \Delta E_{2b} + \Delta E_{3b} + \Delta E_{4b} + \ldots + \Delta E_{nb} \tag{S8}$$

The one-body term, ΔE_{1b}, is the energy of the *i*th monomer in the geometry it adopts in the *n*-mer, E'_i, relative to its isolated equilibrium energy, E_i. In other words, ΔE_{1b} corresponds to the sum of the strain energies of each monomer [eqn (S9a)]. The two-body term correction, ΔE_{2b}, is the sum of all pairwise interaction energies in the *n*-mer [eqn (S9b)]. When truncated at $n \leq 4$, ΔE_{3b} and ΔE_{4b} represent the three- and four-body term corrections, comprising the sum of all three- and four-body interaction energies with all lower-order contributions removed [eqn (S9c and S9d)].

$$\Delta E_{1b} = \sum_i^n \Delta E_{\text{strain,i}} = \sum_i^n E'_i - \sum_i^n E_i \tag{S9a}$$

$$\Delta E_{2b} = \sum_{i<j}^n \Delta E_{ij} = \sum_{i<j}^n (E_{ij} - E'_i - E'_j) \tag{S9b}$$

$$\Delta E_{3b} = \sum_{i<j<k}^n \Delta E_{ijk} = \sum_{i<j<k}^n (E_{ijk} - E'_i - E'_j - E'_k - \Delta E_{ij} - \Delta E_{ik} - \Delta E_{jk}) \tag{S9c}$$

$$\Delta E_{4b} = \sum_{i<j<k<l}^n \Delta E_{ijkl} = \sum_{i<j<k<l}^n (E_{ijkl} - E'_i - E'_j - E'_k - E'_l - \sum_{i<j} \Delta E_{ij} - \sum_{i<j<k} \Delta E_{ijk}) \tag{S9d}$$

2. Physicochemical properties of H_2Ch and HX compounds

Table S1. Boiling temperatures (in K), vaporization enthalpies (in kcal mol^{-1}), and vaporization entropies (in kcal K^{-1} mol^{-1}) at 1 atm for binary hydrides of groups 16 and 17 elements.

Hydride	T_b[4]	$\Delta H_{vap}(T_b)$	$\Delta S_{vap}(T_b)$[a]
**H_2O**	373.14	9.72[10]	0.02604
**H_2S**	212.74	4.46[10]	0.02096
**H_2Se**	232.04	4.71[10]	0.02030
**HF**	292.84	7.26[11]	0.02479
**HCl**	188.34	3.86[10]	0.02049
**HBr**	206.64	4.20[12]	0.02033

[a] Estimated by $\Delta H_{vap}/T_b$.

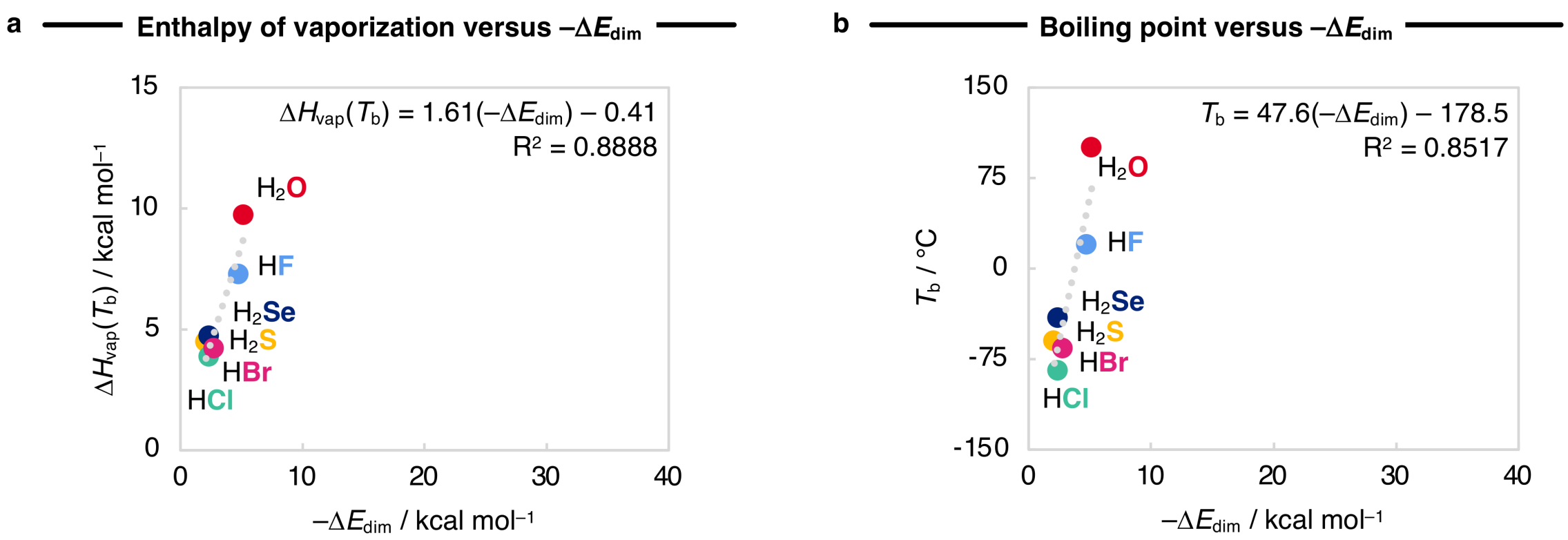

Figure S1. Linear relationship of dimer dissociation energies with (a) enthalpies of vaporization and (b) boiling points for representative binary hydrides of groups 16 and 17 elements of the periodic table. Computed at ZORA-BLYP-D3(BJ)/TZ2P.

3. Stability and polarity of HX model dimers

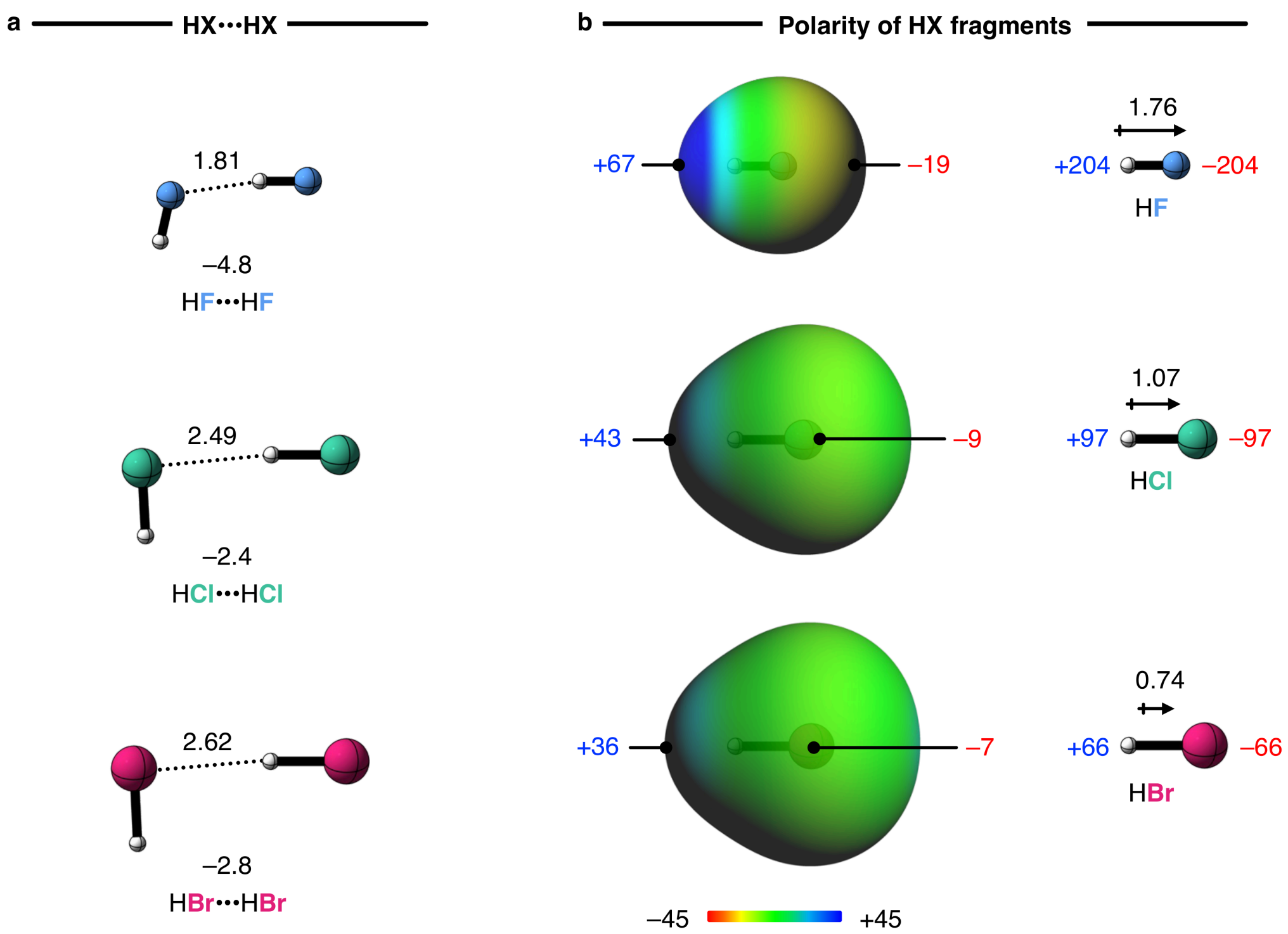

Figure S2. Classical explanation for the abnormal boiling point of HF. (a) Equilibrium geometries (in Å) and dimerization energies (in kcal mol^{-1}) for HX•••HX dimers (X = F, Cl, Br), computed at ZORA-BLYP-D3(BJ)/TZ2P. (b) Molecular electrostatic potentials (in kcal mol^{-1}; isosurfaces at 0.001), dipole moments (in Debye), and Voronoi deformation density charges (in milli-electrons) of HX fragments (X = F, Cl, Br), computed at ZORA-BLYP-D3(BJ)/TZ2P.

4. Activation strain and energy decomposition analyses as a function of the bond distance

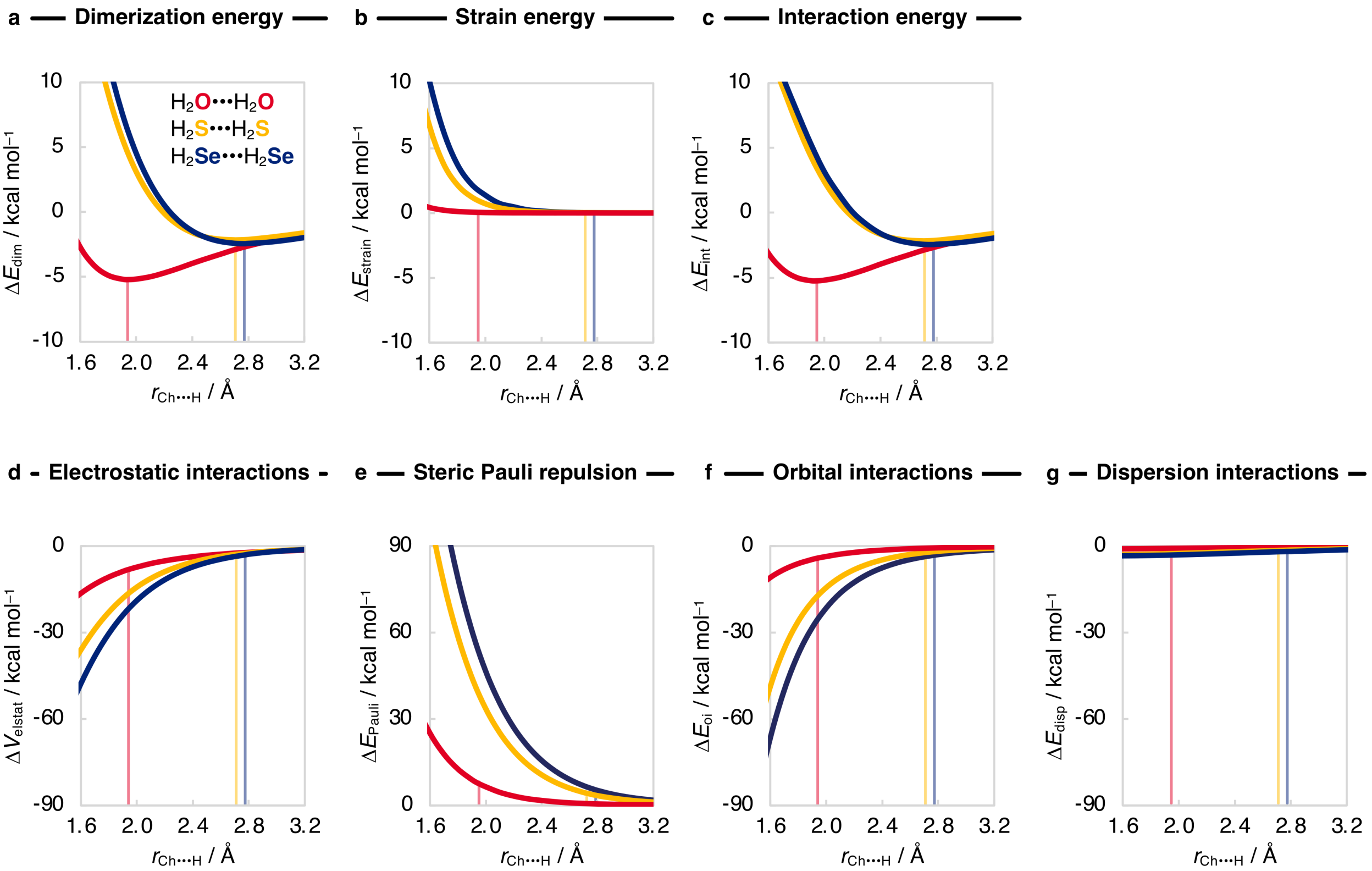

Figure S3. Activation strain and energy decomposition analyses of the hydrogen-bonded $H_2Ch\cdots H_2Ch$ dimers (Ch = O, S, Se) as a function of the Ch•••H distance. (a) Total dimerization energies (in kcal mol^{-1}). (b) Total strain energies of the individual fragments (in kcal mol^{-1}). (c) Interaction energies between distorted fragments (in kcal mol^{-1}). (d) Electrostatic interaction energies between the charge distributions of the H_2Ch fragments. (e) Destabilizing occupied–occupied orbital interaction energy due to the Pauli exclusion principle. (f) Stabilizing donor–acceptor interaction energies responsible for the covalent component of the hydrogen bond. (g) Dispersion correction energies (in kcal mol^{-1}). The vertical lines indicate the equilibrium geometries. Computed at ZORA-BLYP-D3(BJ)/TZ2P.

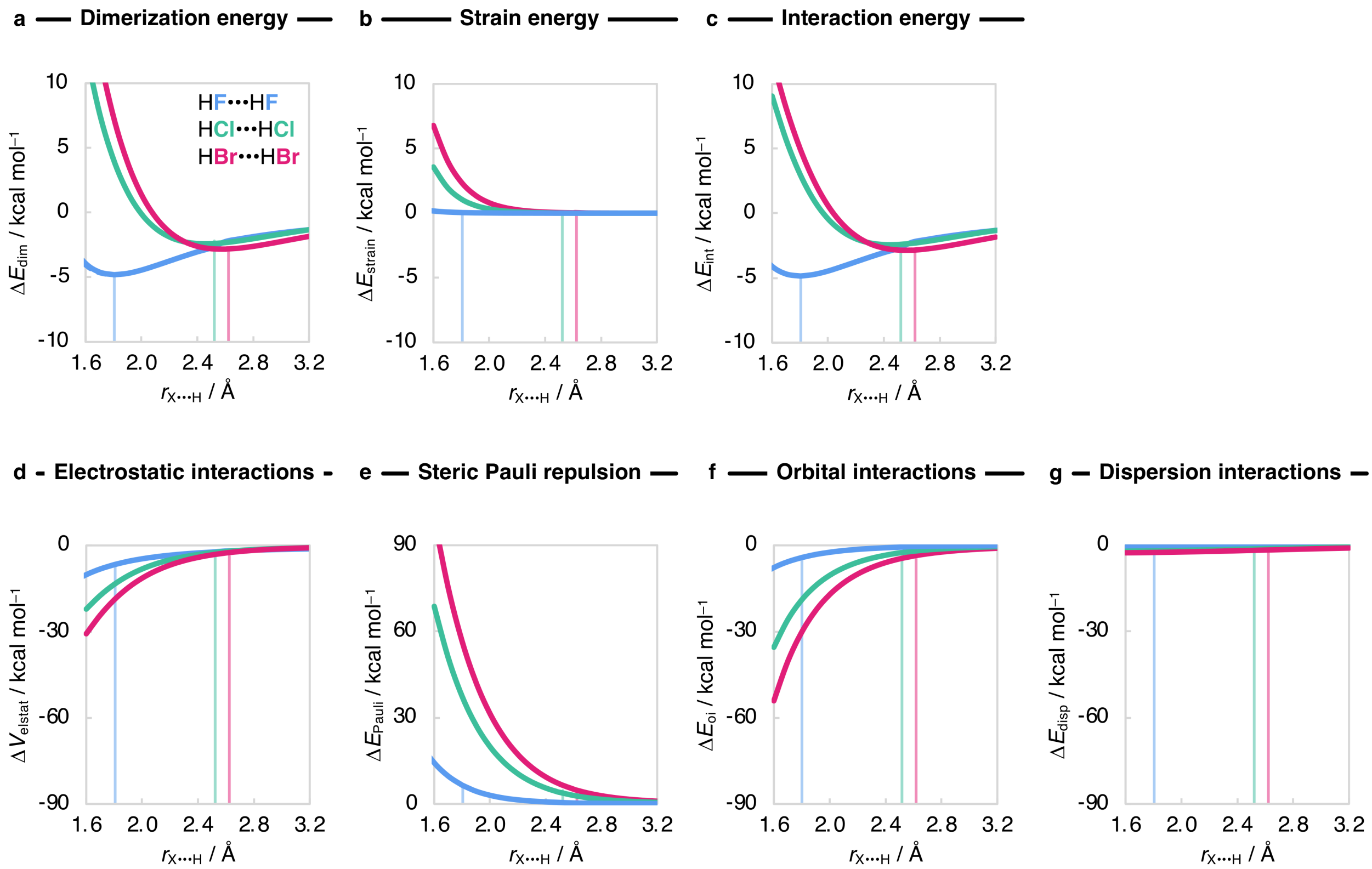

Figure S4. Activation strain and energy decomposition analyses of the hydrogen-bonded HX•••HX dimers (X = F, Cl, Br) as a function of the X•••H distance. (a) Total dimerization energies (in kcal mol^{-1}). (b) Total strain energies of the individual fragments (in kcal mol^{-1}). (c) Interaction energies between distorted fragments (in kcal mol^{-1}). (d) Electrostatic interaction energies between the charge distributions of the H_2Ch fragments. (e) Destabilizing occupied–occupied orbital interaction energy due to the Pauli exclusion principle. (f) Stabilizing donor–acceptor interaction energies responsible for the covalent component of the hydrogen bond. (g) Dispersion correction energies (in kcal mol^{-1}). The vertical lines indicate the equilibrium geometries. Computed at ZORA-BLYP-D3(BJ)/TZ2P.

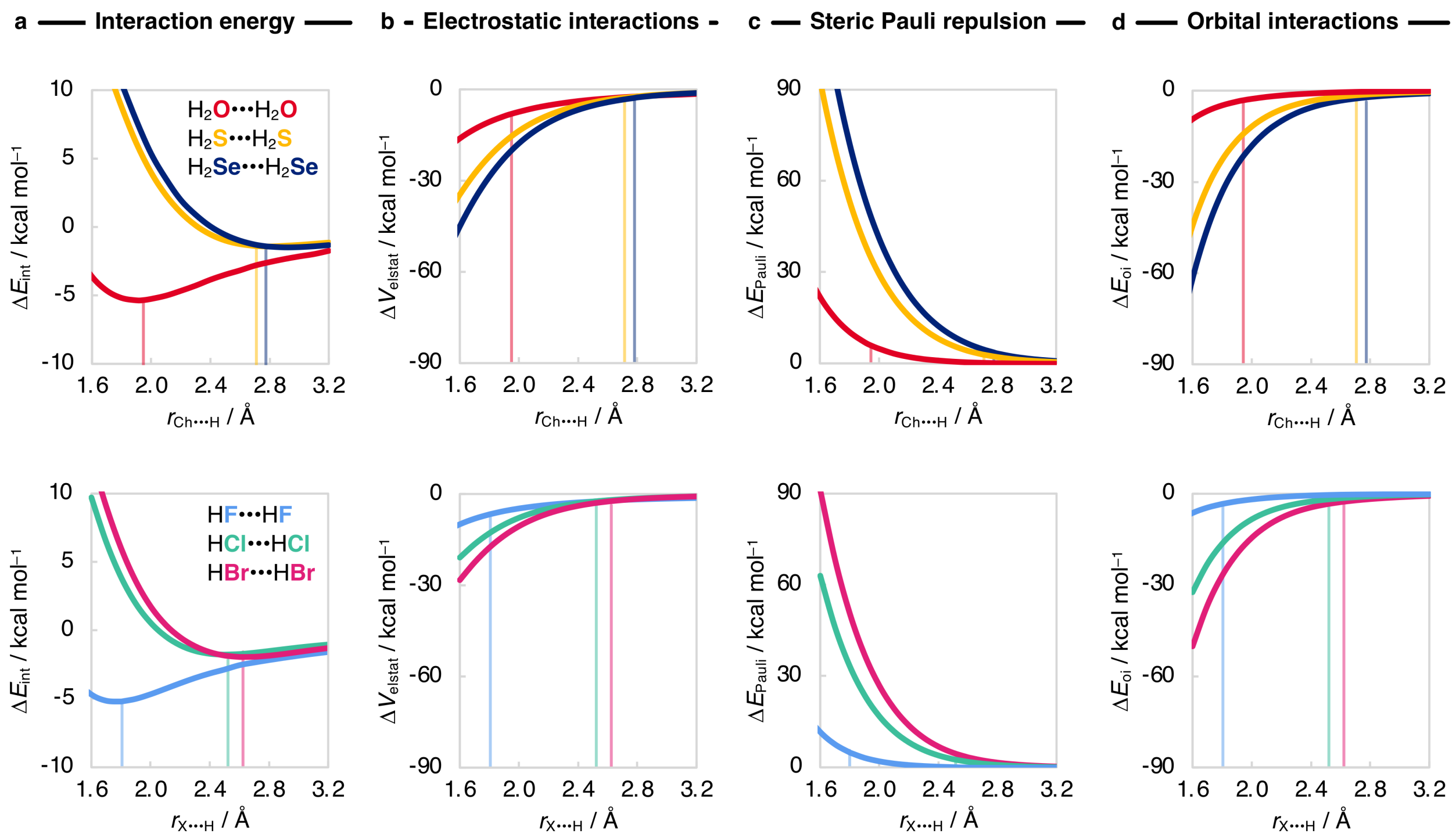

Figure S5. Energy decomposition analyses of the hydrogen-bonded H_2Ch and HX dimers (Ch = O, S, Se; X = F, Cl, Br) as a function of the Ch/X•••H distance. (a) Interaction energies between distorted fragments (in kcal mol^{-1}). (b) Electrostatic interaction energies between the charge distributions of the H_2Ch fragments. (c) Destabilizing occupied–occupied orbital interaction energy due to the Pauli exclusion principle. (d) Stabilizing donor–acceptor interaction energies responsible for the covalent component of the hydrogen bond. The vertical lines indicate the equilibrium geometries. Computed at ZORA-M06-2X/TZ2P//ZORA-BLYP-D3(BJ)/TZ2P.

5. Activation strain, energy decomposition, and quantitative MO analyses at equilibrium geometries

Table S2. Activation strain and energy decomposition analyses. $H_2Ch\cdots H_2Ch$ and $HX\cdots HX$ dimers at their equilibrium geometries (Ch = O, S, Se; X = F, Cl, Br). Bond lengths in Å and energies in kcal mol^{-1}. Computed at ZORA-BLYP-D3(BJ)/TZ2P.

Dimers	$r_{Ch/X\cdots H}$	ΔE	ΔE_{strain}	ΔE_{int}	ΔV_{elstat}	ΔE_{Pauli}	ΔE_{oi}	ΔE_{disp}
**$H_2O\cdots H_2O$**	1.947	–5.20	0.05	–5.25	–8.02	7.64	–4.08	–0.78
**$H_2S\cdots H_2S$**	2.711	–2.15	0.02	–2.17	–2.84	4.37	–2.20	–1.50
**$H_2Se\cdots H_2Se$**	2.775	–2.43	0.02	–2.45	–3.04	5.58	–3.13	–1.86
**$HF\cdots HF$**	1.808	–4.80	0.05	–4.85	–6.59	6.33	–4.13	–0.46
**$HCl\cdots HCl$**	2.486	–2.41	0.01	–2.42	–2.62	4.17	–2.70	–1.27
**$HBr\cdots HBr$**	2.615	–2.81	0.04	–2.85	–2.52	4.86	–3.55	–1.64

Table S3. Quantitative Kohn-Sham molecular orbital analyses. Donor–acceptor orbital energy gap ($\Delta\varepsilon$) in eV. $H_2Ch\cdots H_2Ch$ and $HX\cdots HX$ dimers at their equilibrium geometries and at consistent $r_{Ch/X\cdots H}$ = 2.0 Å. Computed at ZORA-BLYP-D3(BJ)/TZ2P.

		$r_{Ch/X\cdots H,eq}$			$r_{Ch/X\cdots H}$ = 2.0 Å		
**Dimers**	$r_{Ch/X\cdots H,eq}$	$S_{LP-\sigma}$	$S_{LP-\sigma^*}$	$\Delta\varepsilon$	$S_{LP-\sigma}$	$S_{LP-\sigma^*}$	$\Delta\varepsilon$
**$H_2O\cdots H_2O$**	1.947	0.07	0.14	6.3	0.07	0.14	6.3
**$H_2S\cdots H_2S$**	2.711	0.07	0.21	5.3	0.16	0.28	5.2
**$H_2Se\cdots H_2Se$**	2.775	0.09	0.24	4.7	0.17	0.29	4.4
**$HF\cdots HF$**	1.808	0.07	0.18	8.5	0.07	0.18	8.5
**$HCl\cdots HCl$**	2.486	0.08	0.24	6.8	0.14	0.29	6.5
**$HBr\cdots HBr$**	2.615	0.09	0.24	5.8	0.19	0.30	5.4

6. Activation strain and energy decomposition analyses at consistent geometries

Table S4. Activation strain and energy decomposition analyses. $[H_2Ch]_n$ and $[HX]_n$ n-body systems at consistent averaged bond lengths (n = 2, 3, 4; Ch = O, S, Se; X = F, Cl, Br). Bond lengths in Å and energies in kcal mol^{-1}. Computed at ZORA-BLYP-D3(BJ)/TZ2P.

$[H_2Ch]_n$/$[HX]_n$	$\bar{r}_{Ch/X\cdots H}$	ΔE	ΔE_{strain}	ΔE_{int}	ΔV_{elstat}	ΔE_{Pauli}	ΔE_{oi}	ΔE_{disp}
**$[H_2O]_2$**	2.000	–5.16	0.03	–5.19	–7.29	6.47	–3.60	–0.76
**$[H_2S]_2$**	2.000	3.10	0.71	2.39	–14.22	33.54	–14.39	–2.55
**$[H_2Se]_2$**	2.000	4.52	1.37	3.15	–18.86	46.51	–21.46	–3.04
**$[HF]_2$**	2.000	–4.45	0.02	–4.47	–4.65	2.98	–2.39	–0.41
**$[HCl]_2$**	2.000	–0.10	0.33	–0.43	–8.18	19.92	–10.40	–1.77
**$[HBr]_2$**	2.000	1.39	0.80	0.59	–11.38	31.42	–17.11	–2.34
**$[H_2O]_3$**	2.000	–16.19	0.31	–16.50	–22.63	21.39	–12.82	–2.44
**$[H_2S]_3$**	2.000	3.40	4.47	–1.07	–43.80	103.33	–52.59	–8.02
**$[H_2Se]_3$**	2.000	3.03	10.77	–7.74	–57.47	140.72	–81.56	–9.43
**$[HF]_3$**	2.000	–9.86	0.06	–9.92	–9.56	5.83	–5.35	–0.84
**$[HCl]_3$**	2.000	–2.47	0.94	–3.41	–16.99	40.98	–22.94	–4.47
**$[HBr]_3$**	2.000	–2.99	1.11	–4.10	–24.39	64.90	–38.14	–6.46
**$[H_2O]_4$**	2.000	–27.00	0.34	–27.34	–30.93	25.79	–18.78	–3.42
**$[H_2S]_4$**	2.000	–0.25	6.10	–6.35	–58.31	134.39	–70.91	–11.52
**$[H_2Se]_4$**	2.000	–0.76	13.56	–14.32	–76.27	185.62	–109.08	–14.59
**$[HF]_4$**	2.000	–15.73	0.09	–15.82	–14.70	8.71	–8.57	–1.27
**$[HCl]_4$**	2.000	–4.72	1.61	–6.33	–25.13	59.82	–34.92	–6.10
**$[HBr]_4$**	2.000	–2.86	3.97	–6.83	–36.14	97.71	–58.77	–9.64

7. Dimerization energies of fictitious HX dimers

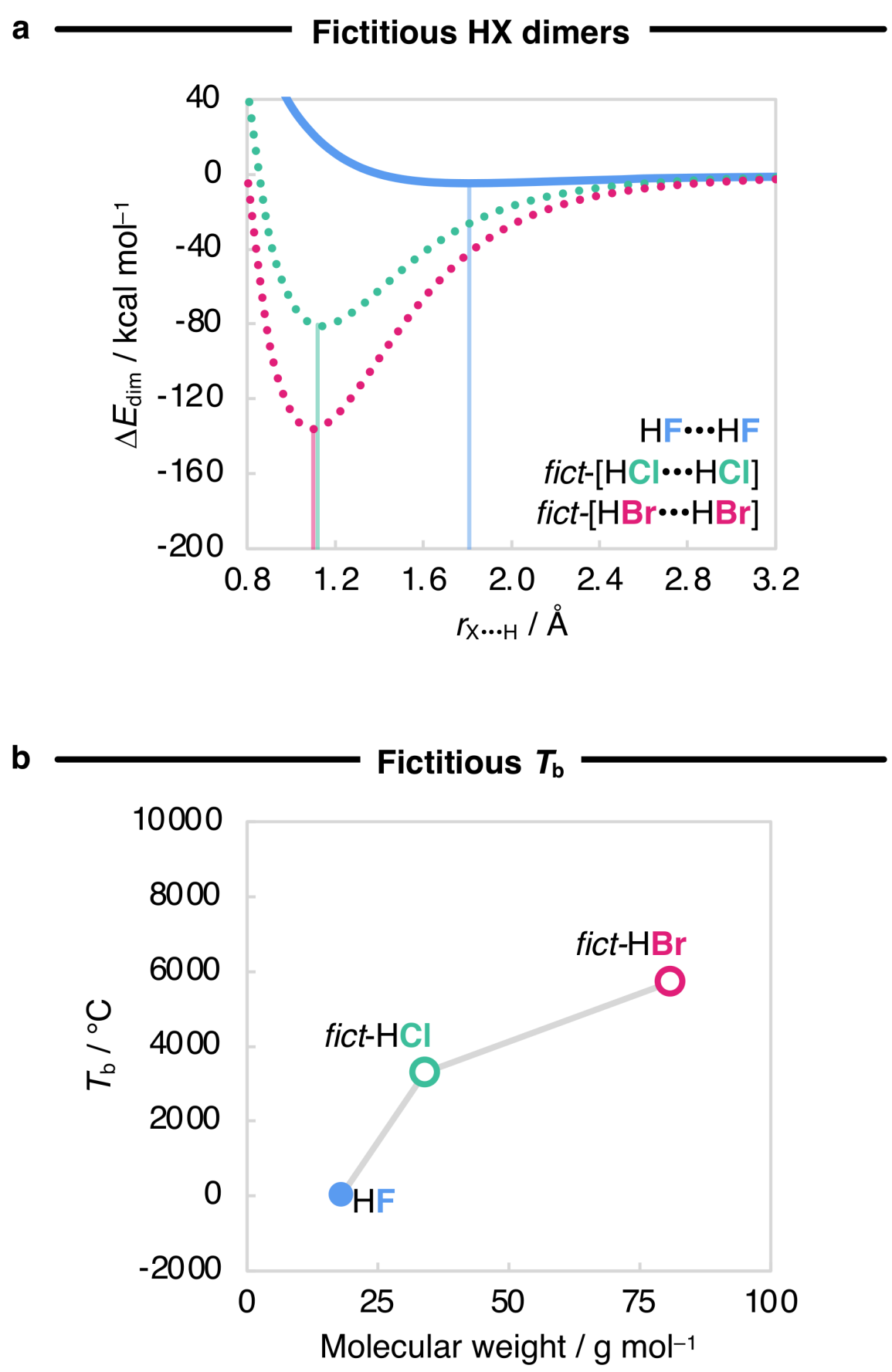

Figure S6. (a) Dimerization energy for the fictitious HX dimers (Ch = F, Cl, Br) and (b) the associated fictitious boiling points obtained using the linear relationship in Fig. 5b. Computed at ZORA-BLYP-D3(BJ)/TZ2P.

8. Cartesian coordinates and electronic energies

Table S5. Cartesian coordinates (in Å), ADF total electronic energies (in kcal mol^{-1}), and the number of imaginary vibrational frequencies for the H_2Ch and HX fragments (Ch = O, S, Se; X = F, Cl Br). Computed at ZORA-BLYP-D3(BJ)/TZ2P.

H_2O
***E* = -317.86**
N_{imag} = 0

O	0.00000000	0.00000000	-0.03284100
H	0.00000000	0.76867800	0.56302200
H	0.00000000	-0.76867800	0.56302200

H_2S
***E* = -245.80**
N_{imag} = 0

S	0.00000000	0.00000000	-0.26266100
H	0.00000000	0.97275000	0.67793200
H	0.00000000	-0.97275000	0.67793200

H_2Se
***E* = -223.26**
N_{imag} = 0

Se	0.00000000	0.00000000	-0.33046800
H	0.00000000	1.05181500	0.71183500
H	0.00000000	-1.05181500	0.71183500

HF
***E* = -178.10**
N_{imag} = 0

F	0.00000000	0.00000000	0.11479200
H	0.00000000	0.00000000	1.05098300

HCl
***E* = -132.86**
N_{imag} = 0

Cl	0.00000000	0.00000000	-0.06412500
H	0.00000000	0.00000000	1.22990000

HBr
***E* = -117.97**
N_{imag} = 0

Br	0.00000000	0.00000000	-0.13439500
H	0.00000000	0.00000000	1.30017000

Table S6. Cartesian coordinates (in Å), ADF total electronic energies (in kcal mol⁻¹), and the number of imaginary vibrational frequencies for the H_2Ch and HX dimers (Ch = O, S, Se; X = F, Cl Br). Computed at ZORA-BLYP-D3(BJ)/TZ2P.

$(H_2O)_2$
***E* = -640.92**
N_{imag} = 0

O	−0.08547400	1.96409300	0.00000000
H	−0.61840300	2.22595300	0.77130000
H	−0.61840300	2.22595300	−0.77130000
H	0.00614600	0.01904000	0.00000000
O	−0.10057200	−0.95592400	0.00000000
H	0.80630500	−1.30455000	0.00000000

$(H_2S)_2$
***E* = -493.75**
N_{imag} = 0

S	1.84247200	−0.14455100	0.00000000
H	1.83098700	0.79504700	0.97370100
H	1.83098700	0.79504700	−0.97370100
H	−0.86822600	−0.13554000	0.00000000
S	−2.21946500	0.01405600	0.00000000
H	−2.41675400	−1.32406000	0.00000000

$(H_2Se)_2$
***E* = -448.95**
N_{imag} = 0

Se	1.91391600	−0.21354500	0.00000000
H	1.88697600	0.82765500	1.05286000
H	1.88697600	0.82765500	−1.05286000
H	−0.85798500	−0.07852200	0.00000000
Se	−2.34097600	0.05526400	0.00000000
H	−2.48890700	−1.41850700	0.00000000

$(HF)_2$
***E* = -361.00**
N_{imag} = 0

F	−0.21304400	−1.06051500	0.00000000
H	0.67421800	−1.36981400	0.00000000
H	−0.28590100	0.74642400	0.00000000
F	−0.17527300	1.68390500	0.00000000

$(HCl)_2$
***E* = -268.13**
N_{imag} = 0

Cl	−0.32120600	−2.06657800	0.00000000
H	0.96005300	−2.26288700	0.00000000
H	−0.07061900	0.40637000	0.00000000
Cl	0.19273900	1.68021900	0.00000000

$(HBr)_2$
***E* = -238.75**
N_{imag} = 0

Br	−0.56730200	−2.42152300	0.00000000
H	0.84495100	−2.68013000	0.00000000
H	−0.10862700	0.15260300	0.00000000
Br	0.21920000	1.55923600	0.00000000

Table S7. Cartesian coordinates (in Å), ADF total electronic energies (in kcal mol^{-1}), and the number of imaginary vibrational frequencies (in cm^{-1}) for the H_2Ch and HX trimers (Ch = O, S, Se; X = F, Cl Br). Computed at ZORA-BLYP-D3(BJ)/TZ2P.

$(H_2O)_3$
***E* = -970.18**
N_{imag} = 0

O	0.97107017	0.00035813	−2.63358122
H	1.80912800	0.08278414	−2.11537069
H	0.91743664	0.80379196	−3.17776476
O	0.00000000	0.00000000	0.00000000
H	0.04034364	−0.02829411	−0.98817250
H	−0.45253973	−0.81772819	0.26728156
O	2.76689641	0.00247592	−0.45908538
H	1.89043818	−0.00000000	0.00000000
H	3.23952356	0.78017668	−0.11824337

$(H_2S)_3$
***E* = -744.39**
N_{imag} = 0

S	1.58125314	−0.38226330	−3.63326771
H	2.59084426	−0.14747875	−2.74978925
H	1.52134296	0.91937243	−3.99585122
S	0.00000000	0.00000000	0.00000000
H	0.30947652	−0.13620450	−1.32005232
H	−0.10179091	−1.33892137	0.16455776
S	3.93247978	0.07599767	−0.45425494
H	2.64995337	0.00000000	0.00000000
H	3.93514053	1.42530978	−0.36069844

$(H_2Se)_3$
***E* = -677.69**
N_{imag} = 0

Se	1.71948886	−0.05457951	−3.75306700
H	2.77122347	0.03059817	−2.69330877
H	1.55318788	1.41628291	−3.73372022
Se	−0.00000000	0.00000000	0.00000000
H	0.36425155	−0.03036448	−1.45028066
H	0.03466953	−1.47885445	0.05299859
Se	4.11619245	0.01155368	−0.38456782
H	2.67079862	−0.00000000	−0.00000000
H	4.09056078	1.48775824	−0.48912078

$(HF)_3$, C_s symmetry enforced
***E* = -545.63**
N_{imag} = 1 (48.4i)

F	0.95233343	0.00000000	0.00000000
H	0.00000000	0.00000000	0.00000000
F	−1.70565639	−0.14865302	0.00000000
H	−2.04402052	−1.02621039	0.00000000
F	1.97298688	2.48922868	0.00000000
H	1.65430144	1.59719407	0.00000000

$(HCl)_3$, C_s symmetry enforced
***E* = -403.85**
N_{imag} = 0

Cl	1.30527986	0.00000000	0.00000000
H	0.00000000	0.00000000	0.00000000
Cl	−2.40235697	−0.43539057	0.00000000
H	−2.25319788	−1.72246113	0.00000000
Cl	2.09195630	3.64273497	0.00000000
H	1.77833334	2.37839998	0.00000000

$(HBr)_3$, C_s symmetry enforced
***E* = -361.18**
N_{imag} = 0

Br	1.45152352	0.00000000	0.00000000
H	0.00000000	0.00000000	0.00000000
Br	−2.40824196	0.61668353	0.00000000
H	−2.96097056	−0.71118906	0.00000000
Br	−0.13327361	3.62029714	0.00000000
H	0.69114286	2.42924319	0.00000000

Table S8. Cartesian coordinates (in Å), ADF total electronic energies (in kcal mol^{-1}), and the number of imaginary vibrational frequencies (in cm^{-1}) for the H_2Ch and HX tetramers (Ch = O, S, Se; X = F, Cl Br). Computed at ZORA-BLYP-D3(BJ)/TZ2P.

$(H_2O)_4$
***E* = -1301.37**
N_{imag} = 0

O	-1.31819600	1.40939100	0.03864100
H	-1.77443500	1.77401200	-0.73794300
H	-1.47641800	0.42326200	0.00750800
O	-1.40939100	-1.31819600	-0.03864100
H	-1.77401200	-1.77443500	0.73794300
H	-0.42326200	-1.47641800	-0.00750800
O	1.31819600	-1.40939100	0.03864100
H	1.47641800	-0.42326200	0.00750800
H	1.77443500	-1.77401200	-0.73794300
O	1.40939100	1.31819600	-0.03864100
H	1.77401200	1.77443500	0.73794300
H	0.42326200	1.47641800	-0.00750800

$(H_2S)_4$
***E* = -994.37**
N_{imag} = 0

S	-1.86584600	1.71236400	0.76248600
H	-2.40665900	2.27832200	-0.34038200
H	-1.90632400	0.46415800	0.20729900
S	-1.71236400	-1.86584600	-0.76248600
H	-2.27832200	-2.40665900	0.34038200
H	-0.46415800	-1.90632400	-0.20729900
S	1.86584600	-1.71236400	0.76248600
H	1.90632400	-0.46415800	0.20729900
H	2.40665900	-2.27832200	-0.34038200
S	1.71236400	1.86584600	-0.76248600
H	2.27832200	2.40665900	0.34038200
H	0.46415800	1.90632400	-0.20729900

$(H_2Se)_4$
***E* = -906.34**
N_{imag} = 0

Se	-1.49016400	1.21475800	1.54749700
H	-2.25706900	2.29887100	0.88997100
H	-1.68923000	0.32359200	0.36633400
Se	-1.21475800	-1.49016400	-1.54749700
H	-2.29887100	-2.25706900	-0.88997100
H	-0.32359200	-1.68923000	-0.36633400
Se	1.49016400	-1.21475800	1.54749700
H	1.68923000	-0.32359200	0.36633400
H	2.25706900	-2.29887100	0.88997100
Se	1.21475800	1.49016400	-1.54749700
H	2.29887100	2.25706900	-0.88997100
H	0.32359200	1.68923000	-0.36633400

$(HF)_4$, C_s symmetry enforced
***E* = -731.25**
N_{imag} = 2 (10.8i, 48.5i)

F	0.95540959	0.00000000	0.00000000
H	0.00000000	0.00000000	0.00000000
F	-1.67520312	-0.10120205	0.00000000
H	-2.06314901	-0.95865077	0.00000000
F	4.72445116	2.30123249	0.00000000
H	3.77622490	2.34947544	0.00000000
F	2.05731925	2.35392816	0.00000000
H	1.66920724	1.47782440	0.00000000

$(HCl)_4$, C_s symmetry enforced
***E* = -540.19**
N_{imag} = 0

Cl	1.30802429	0.00000000	0.00000000
H	0.00000000	0.00000000	0.00000000
Cl	-2.35706739	-0.01710046	0.00000000
H	-2.47976243	-1.30805695	0.00000000
Cl	4.86873186	3.56278617	0.00000000
H	3.56676875	3.65323158	0.00000000
Cl	1.18005284	3.60847505	0.00000000
H	1.26159143	2.30036652	0.00000000

$(HBr)_4$, C_s symmetry enforced
***E* = -482.50**
N_{imag} = 1 (6.5i)

Br	1.45161260	0.00000000	0.00000000
H	0.00000000	0.00000000	0.00000000
Br	-2.45916080	0.42790385	0.00000000
H	-2.81303018	-0.96450993	0.00000000
Br	4.85570301	2.15838322	0.00000000
H	3.66218201	2.97800199	0.00000000
Br	1.26216200	3.84561261	0.00000000
H	1.24742849	2.38854809	0.00000000

9. References

4 D. R. Stull, "Vapor Pressure of Pure Substances. Organic and Inorganic Compounds," *Ind. Eng. Chem.*, 1947, **39**, 517-550, DOI: 10.1021/ie50448a022.

6 J. N. Israelachvili. *Intermolecular and surface forces*, 3rd ed.; Academic press, 2011.

7 P. W. Atkins and J. de Paula, Physical Chemistry, 9th ed., Oxford University Press, 2010.

8 F. Jensen, Introduction to Computational Chemistry, 2nd ed.; Wiley, 2007

10 CRC Handbook of Chemistry and Physics, 84th ed.; Lide, D. R., Ed.; CRC Press: Boca Raton, Florida, 2003.

11 C. E. Vanderzee and W. W. Rodenburg, "Gas imperfections and thermodynamic excess properties of gaseous hydrogen fluoride," *J. Chem. Thermodyn.*, 1970, 2, 461-478, DOI: 10.1016/0021-9614(70)90097-2.

12 W. F. Giauque and R. Wiebe, "THE HEAT CAPACITY OF HYDROGEN BROMIDE FROM 15°K. TO ITS BOILING POINT AND ITS HEAT OF VAPORIZATION. THE ENTROPY FROM SPECTROSCOPIC DATA," *J. Am. Chem. Soc.*, 1928, 50, 2193-2202, DOI: 10.1021/ja01395a018.

19 R. M. Richard, K. U. Lao and J. M. Herbert, "Understanding the many-body expansion for large systems. I. Precision considerations," *J. Chem. Phys.*, 2014, 141, 014108, DOI: 10.1063/1.4885846.

25 F. M. Bickelhaupt and E. J. Baerends, Reviews in Computational Chemistry. *Kohn-Sham Density Functional Theory: Predicting and Understanding Chemistry*; K. B. Lipkowitz, D. B. Boyd, Wiley, 2000, pp. 1–86. DOI: 10.1002/9780470125922.ch1.

38 AMS2023.101, SCM Theoretical Chemistry; Vrije Universiteit: Amsterdam, The Netherlands; http://www.scm.com.

39 G. te Velde, F. M. Bickelhaupt, E. J. Baerends, et. al., "Chemistry with ADF," *J. Comput. Chem.*, 2001, 22, 931-967, DOI: 10.1002/jcc.1056.

40 E. J. Baerends, N. F. Aguirre, N. D. Austin, et. al., "The Amsterdam Modeling Suite," *J. Chem. Phys.*, 2025, 162, 162501/1-11, DOI: 10.1063/5.0258496.

41 A. D. Becke, "Density-functional exchange-energy approximation with correct asymptotic behavior," *Phys. Rev. A*, 1998, 38, 3098–3100, DOI: 10.1103/PhysRevA.38.3098.

42 C. Lee, W. Yang and R. G. Parr, "Development of the Colle-Salvetti correlation-energy formula into a functional of the electron density," *Phys. Rev. B*, 1988, 37, 785–789, DOI: 10.1103/PhysRevB.37.785.

43 S. Grimme, J. Antony, S. Ehrlich and H. Krieg, "A consistent and accurate ab initio parametrization of density functional dispersion correction (DFT-D) for the 94 elements H-Pu," *J. Chem. Phys.*, 2010, 132, 154104, DOI: 10.1063/1.3382344.

44 S. Grimme, S. Ehrlich and L. Goerigk, "Effect of the damping function in dispersion corrected density functional theory," *J. Comput. Chem.*, 2011, 32, 1456–1465, DOI: 10.1002/jcc.21759.

45 E. R. Johnson and A. D. Becke, "Exchange-hole dipole moment and the dispersion interaction: High-order dispersion coefficients," *J. Chem. Phys.*, 2005, 123, 024101, DOI: 10.1063/1.2139668.

46 E. van Lenthe, E. J. Baerends and J. G. Snijders, "Relativistic total energy using regular approximations," *J. Chem. Phys.*, 1994, 101, 9783–9792, DOI: 10.1063/1.467943.

47 P. Vermeeren, L. P. Wolters, G. Paragi and C. Fonseca Guerra, "Cooperative Self-Assembly in Linear Chains Based on Halogen Bonds," *ChemPlusChem*, 2010, 86, 812–819, DOI: 10.1002/cplu.202100093.

48 N. Mardirossian and M. Head-Gordon, "Thirty years of density functional theory in computational chemistry: an overview and extensive assessment of 200 density functionals," *Mol. Phys.*, 2017, 115, 2315–2372, DOI: 10.1080/00268976.2017.1333644.

49 E. C. Mitchell, L. de Azevedo Santos, P. Vermeeren, et. al., "Hydrogen Bond Benchmark: Focal-Point Analysis and Assessment of DFT Functionals," *J. Comput. Chem.*, 2025, 46, e70265, DOI: 10.1002/jcc.70265.

50 E. van Lenthe and E. J. Baerends, "Optimized Slater-type basis sets for the elements 1–118," *Comput. Chem.*, 2003, 24, 1142-1156, DOI: 10.1002/jcc.10255.

51 M. Franchini, P. H. T. Philipsen, E. van Lenthe and L. Visscher, "Accurate Coulomb Potentials for Periodic and Molecular Systems through Density Fitting," *J. Chem. Theory Comput.*, 2014, 10, 1994–2004, DOI: 10.1021/ct500172n.

52 M. Franchini, P. H. T. Philipsen and L. Visscher, "The Becke Fuzzy Cells Integration Scheme in the Amsterdam Density Functional Program Suite," *J. Comput. Chem.*, 2013, 34, 1819–1827, DOI: 10.1002/jcc.23323.

53 X. Sun, T. Soini, L. P. Wolters, W.-J. van Zeist, C. Fonseca Guerra, T. A. Hamlin and F. M. Bickelhaupt, PyFrag 2019, Vrije Universiteit Amsterdam, The Netherlands.

54 X. Sun, T. M. Soini, J. Poater, T. A. Hamlin and F. M. Bickelhaupt, "PyFrag 2019–Automating the exploration and analysis of reaction mechanisms," *J. Comput. Chem.*, 2019, 40, 2227–2233, DOI: 10.1002/jcc.25871.

55 Y. Zhao and D. G. Truhlar, "A new local density functional for main-group thermochemistry, transition metal bonding, thermochemical kinetics, and noncovalent interactions," *J. Chem. Phys.*, 2006, 125, 194101, DOI: 10.1063/1.2370993.

56 Y. Zhao and D. G. Truhlar, "The M06 suite of density functionals for main group thermochemistry, thermochemical kinetics, noncovalent interactions, excited states, and transition elements: two new functionals and systematic testing of four M06-class functionals and 12 other functionals," *Theor. Chem. Acc.*, 2008, 120, 215, DOI: 10.1007/s00214-007-0310-x.

57 C. Nieuwland, P. Vermeeren, F. M. Bickelhaupt and C. Fonseca Guerra, "Understanding chemistry with the symmetry-decomposed Voronoi deformation density charge analysis," *J. Comput. Chem.*, 2023, 44, 2108–2119, DOI: 10.1002/jcc.27184.
